# FOXQ1 activates GLT8D2 to enhance CCL2 N-glycosylation and promote prostate cancer bone metastasis

**DOI:** 10.1038/s41419-026-09046-9

**Published:** 2026-07-01

**Authors:** Zhiyue Xie, Nan Peng, Linglan Zhao, Chuqian Zhen, Xian-Lu Song, Yaying Hong, Qu Li, Tao Xie, Tianming Peng, Xianzi Zeng, Jinpeng Cen, Shengdong Ge, Xiaofeng Liu, Ninghan Feng, Mingkun Chen, Shan-Chao Zhao

**Affiliations:** 1 Department of Urology, Nanfang Hospital, Southern Medical University, Guangzhou, China; 2 Department of Urology, The Fifth Affiliated Hospital, Southern Medical University, Guangzhou, China; 3 Department of Urology, The First Affiliated Hospital, Wenzhou Medical University, Wenzhou, China; 4 Department of Radiotherapy, Guangzhou Institute of Cancer Research, The Affiliated Cancer Hospital, Guangzhou Medical University, Guangzhou, China; 5 Department of Urology, Xiang’an Hospital, Xiamen University, Xiamen, China; 6 Guangdong Key Laboratory of Urology, Guangdong Engineering Research Center of Urinary Minimally Invasive Surgery Robot and Intelligent Equipment, Department of Urology, The First Affiliated Hospital of Guangzhou Medical University, Guangzhou, China; 7 Department of Urology, Jiangnan University Medical Center, Wuxi, China; 8 Wuxi School of Medicine, Jiangnan University, Wuxi, China; 9 Department of Urology, Affiliated Wuxi No.2 Hospital, Nanjing Medical University, Wuxi, China; 10 Department of Urology, The Fourth Affiliated Hospital of Guangzhou Medical University, Guangzhou, China

**Keywords:** Prostate cancer, Bone metastases, Glycosylation, Transcriptional regulatory elements

## Abstract

Prostate cancer (PCa) is a common malignancy in men, and bone metastasis is a leading cause of mortality in advanced-stage PCa. This study aims to identify critical genes involved in PCa bone metastasis, exploring biomarkers for prognosis and precision treatment. Forkhead Box Q1 (FOXQ1) was identified as a potential key gene through screening of public databases, and was found to be markedly upregulated in bone metastatic PCa compared to primary PCa. FOXQ1 promotes PCa cell proliferation and metastasis while inhibiting apoptosis. Additionally, FOXQ1 recruits macrophages, promotes M2 polarization, and enhances osteoclast differentiation in the tumor microenvironment. Mechanistically, FOXQ1 activates the transcription of Glycosyltransferase 8 Domain Containing 2 (GLT8D2) by directly binding to its promoter, and GLT8D2 upregulates the expression of C-C Motif Chemokine Ligand 2 (CCL2) by enhancing its N-glycosylation, thereby promoting PCa bone metastasis. Collectively, these findings establish FOXQ1 as a key regulator of PCa bone metastasis through the GLT8D2/CCL2 axis, and suggest that targeting this pathway may hold therapeutic promise for bone metastatic PCa.

## Background

Prostate cancer (PCa) is the second most common cancer and the fifth leading cause of cancer-related deaths in men globally [1, 2]. Statistical data indicate that bone metastasis occurs in 70% of advanced PCa cases [3, 4], which represents the primary cause of mortality in PCa patients [5]. There are currently no effective treatments for bone metastatic PCa. Therefore, research is urgently needed to deepen our understanding of PCa bone metastasis. Previous studies have demonstrated that epithelial-mesenchymal transition (EMT) and the Wnt signaling pathway play crucial roles in PCa bone metastasis. EMT initiates tumor metastasis by inducing a migratory phenotype in PCa cells [6–8], while the Wnt signaling pathway is involved in multiple stages of tumor progression, including tumor growth, metastasis, bone colonization, dormancy, and reactivation after dormancy [9]. Additionally, the Wnt pathway is associated with abnormal bone metabolism in PCa bone metastatic lesions [10]. This study aims to identify key genes that promote PCa bone metastasis.

The Forkhead Box (FOX) protein family is one of the largest families of transcription factors in mammals, comprising 50 human-derived proteins and 44 mouse-derived proteins [11]. A common feature of these proteins is the highly conserved FOX domain, which binds to specific DNA sequences and regulates the transcription of target genes [12].

FOXQ1 was identified as a key protein regulating PCa bone metastasis in this study. Functionally, the FOX domain in the center of FOXQ1 acts as a transactivation domain, recruiting transcriptional co-factors to regulate the transcription of target genes [13, 14]. Previous studies have shown that FOXQ1 is associated with poor prognosis in various malignancies, such as colorectal cancer [15], pancreatic cancer [16], and esophageal cancer [17], and is involved in tumor metastasis, acquired drug resistance, and angiogenesis [18]. However, the role of FOXQ1 in tumor bone metastasis remains unclear. Research on FOXQ1 in PCa is limited. It was suggested that FOXQ1 promotes PCa cell proliferation and inhibits apoptosis [19], and is associated with the development of castration-resistant PCa (CRPC) [20]. Therefore, we aim to investigate the role and molecular mechanisms of FOXQ1 in PCa bone metastasis.

In this study, we utilized public databases to screen for potential key proteins associated with PCa bone metastasis and explored their roles and molecular mechanisms in PCa bone metastasis. FOXQ1 was identified as the key protein. Our results demonstrated that FOXQ1 promotes PCa cell proliferation and migration, and inhibits tumor cell apoptosis. Furthermore, FOXQ1 enhances the recruitment of macrophages, M2 polarization, and osteoclast differentiation within the tumor microenvironment. Mechanistically, we found that FOXQ1 activates the transcription of GLT8D2, which in turn promotes the expression of C-C Motif Chemokine Ligand 2 (CCL2) by enhancing its N-glycosylation. Overall, our findings provide evidence that the FOXQ1/GLT8D2/CCL2 axis contributes to the promotion of PCa bone metastasis.

## Methods

### Clinical samples

Tissues of benign prostatic hyperplasia (BPH), primary PCa, and bone metastatic PCa were obtained from patients treated at the Nanfang Hospital of Southern Medical University and were pathologically confirmed. The age of the patients ranged from 45 to 80 years. These patients did not receive chemotherapy or radiotherapy prior to surgery. Within 30 min of resection, a portion of each tissue specimen was snap-frozen in liquid nitrogen for RNA extraction, and the remaining portion was fixed in 4% paraformaldehyde and paraffin-embedded. Written informed consent (INFORMED) was obtained from all patients prior to surgery. The study was approved by the Ethics Committee of the Nanfang Hospital of Southern Medical University (No. NFEC-202510-K7). All procedures of the research were conducted in accordance with the Declaration of Helsinki.

### Cell culture

The prostate cell lines RWPE-1, C4-2, 22RV1, LNCaP, C4-2B, DU145, PC3, and monocytic cell lines THP-1 and U937 were sourced from the Cell Bank of the Chinese Academy of Sciences. Bone marrow macrophages (BMMs) were isolated from the tibiae and femora of C57BL/6 mice. Cells were cultured at 37 °C with 5% CO_2_ in RPMI 1640 (Gibco, USA), K-SFM (Gibco, USA), F-12K (Gibco, USA), and DMEM (Gibco, USA), supplemented with 10% fetal bovine serum (FBS) (Procell, China) and a penicillin-streptomycin-amphotericin B solution (MeilunBio, China). The PCa cell supernatants were filtered using a 0.45 μm filter (Merck Millipore, Germany) and concentrated using a 3 kDa ultrafiltration tube (Merck Millipore, Germany). The concentrated supernatant was mixed 1:1 with RPMI 1640 to form a co-culture medium for macrophage recruitment or polarization, which was used in transwell assays or flow cytometry analysis. The supernatant was also mixed 1:1 with osteoclast induction medium (Puhe Bio, China) to create a co-culture medium for osteoclast differentiation. The osteoclast differentiation was observed using a Tartrate-Resistant Acid Phosphatase (TRAP) kit (Solarbio, China). Anti-CCL2 (Invitrogen, USA) was used at a concentration of 10 μg/mL.

We constructed three siRNAs targeting FOXQ1 and three shRNAs targeting FOXQ1 and GLT8D2, which were packaged into lentivirus. The plasmid sequences targeting these genes are listed in Table S1. Lentiviruses for stable FOXQ1 overexpression and luciferase-containing constructs were also generated. The siRNAs were transfected into PCa cells using Lipofectamine 3000 (Invitrogen, USA). Lentiviruses were transfected using Polybrene (Tsingke Bio, China) and stable cell lines were selected with antibiotics. All plasmids were designed and constructed by Tsingke Bio (China).

### Animals

BALB/c-nude and C57BL/6 mice were housed in a barrier facility with high-efficiency particulate air (HEPA) filtration and were fed autoclaved laboratory rodent chow. All procedures followed animal ethics guidelines and were approved by the Animal Ethics and Use Committee of Southern Medical University (No. IACUC-LAC-20250927-001).

PCa cells transfected with luciferase (1 × 10⁶ cells per mouse in 100 μL PBS) were injected into the left cardiac ventricle of male BALB/c-nude mice (4-6 weeks old) to establish an arterial dissemination model. Two weeks after injection, in vivo imaging was performed to monitor systemic tumor colonization.

PCa cells (5 × 10^6^ cells per mouse in 100 μL PBS) were subcutaneously injected into the left axilla of male BALB/c-nude mice (4–6 weeks old) to establish subcutaneous tumor models. After 20 days, the mice were euthanized via cervical dislocation, and the tumors were harvested for histological analysis. Tumor volume was calculated using the formula: Volume (mm³) = (width² × length) / 2.

PCa cells (5 × 10^5^ cells per mouse in 100 μL PBS) were intravenously injected into male BALB/c-nude mice (4–6 weeks old) to establish a tail vein lung metastasis model. In vivo imaging was performed after a certain period of time, and the mice were euthanized as described above. The lungs were harvested and examined for metastatic lesions.

PCa cells transfected with luciferase (5 × 10^5^ cells per mouse in 20 μL PBS) were injected into the tibiae of male BALB/c-nude mice (4–6 weeks old) to establish a tibial tumor model, using a within-subject design. In the CCL2 blockade experiment, 5 μg/10μL of anti-CCL2 (Invitrogen, USA) was administered intratibially every 2 days, while the control group received an equal volume of PBS. In vivo imaging was performed after a certain period of time, followed by euthanasia. Tibiae were analyzed using micro-CT to assess bone mineral density at 50 layers starting from the growth plate.

### Bioinformatics analysis

Data relevant to prad_su2c_2019 and prad_fhcrc were obtained from cBioPortal (https://www.cbioportal.org/). PCa-related data from The Cancer Genome Atlas (TCGA, https://portal.gdc.cancer.gov/) were accessed via UCSC Xena (http://xenabrowser.net/datapages/?cohort). The two RNA-seq datasets (TPM) were merged, and batch effects were removed using the ComBat algorithm implemented in the sva R package, an empirical Bayes method widely used for correcting technical variations in high-throughput gene expression studies [21]. EMT-related gene data were retrieved from dbEMT 2.0 (http://dbemt.bioinfo-minzhao.org/index.html), and Wnt signaling pathway genes were identified from previous research [22].

To identify molecular subtypes associated with the Wnt pathway, consensus clustering was performed using the ConsensusClusterPlus R package. The optimal number of clusters (k) was determined by evaluating k from 2 to 10. The clustering procedure was repeated 1000 times with 80% item resampling to ensure robustness and reproducibility. The final cluster heatmap was generated using the pheatmap function.

TIMER (https://cistrome.shinyapps.io/timer/) and GEPIA (http://gepia2.cancer-pku.cn/) were used for immune cell infiltration and gene expression correlation analyses. Single-cell markers were analyzed using CellMarker 2.0 (http://117.50.127.228/CellMarker/), and potential downstream target genes of FOXQ1 were predicted using GTRD (http://gtrd.biouml.org/). Binding sites of FOXQ1 on the GLT8D2 promoter were predicted using JASPAR (https://jaspar.elixir.no/) and GeneCards (https://www.genecards.org/). Structural data for GLT8D2 and CCL2 were obtained from Uniprot (https://www.uniprot.org/) and PDB (https://www.rcsb.org/), and molecular docking models were predicted using GRAMM (http://gramm.compbio.ku.edu/) and PDBePISA (https://www.ebi.ac.uk/msd-srv/prot_int/). Single-cell RNA sequencing data were analyzed in RStudio using R packages including Seurat, SingleR, ggplot2, monocle, monocle3, CellChat, and clusterProfiler. Target cell populations were extracted from the global dataset, re-clustered using the same Seurat workflow with a moderately increased resolution parameter to obtain finer subclusters, and subsequently annotated based on the CellMarker 2.0 database. RNA-seq was conducted by Tsingke Biotech.

### Statistical analysis

Statistical analyses were performed using Excel 2016 or GraphPad Prism 8.0 software. Chi-square (c^2^) tests were used to compare protein expression levels of FOXQ1, GLT8D2, and CCL2 in BPH, primary PCa, and bone metastatic PCa tissues. For in vitro and molecular biology studies, pairwise comparisons were performed using t-tests, and comparisons between multiple groups were performed using analysis of variance (ANOVA). *P* < 0.05 was considered statistically significant (*P < 0.05, **P < 0.01, ***P < 0.001, ns for no significant difference).

The remaining experimental methods and materials are presented in the Supplementary Materials.

### Ethics approval and consent to participate

The study was approved by the Ethics Committee of the Nanfang Hospital of Southern Medical University (No. NFEC-202510-K7). All procedures of the research were conducted in accordance with the Declaration of Helsinki. All procedures followed animal ethics guidelines and were approved by the Animal Ethics and Use Committee of Southern Medical University (No. IACUC-LAC-20250927-001).

## Results

### FOXQ1 is identified as a potential key gene in PCa bone metastasis

**Fig. 1:**
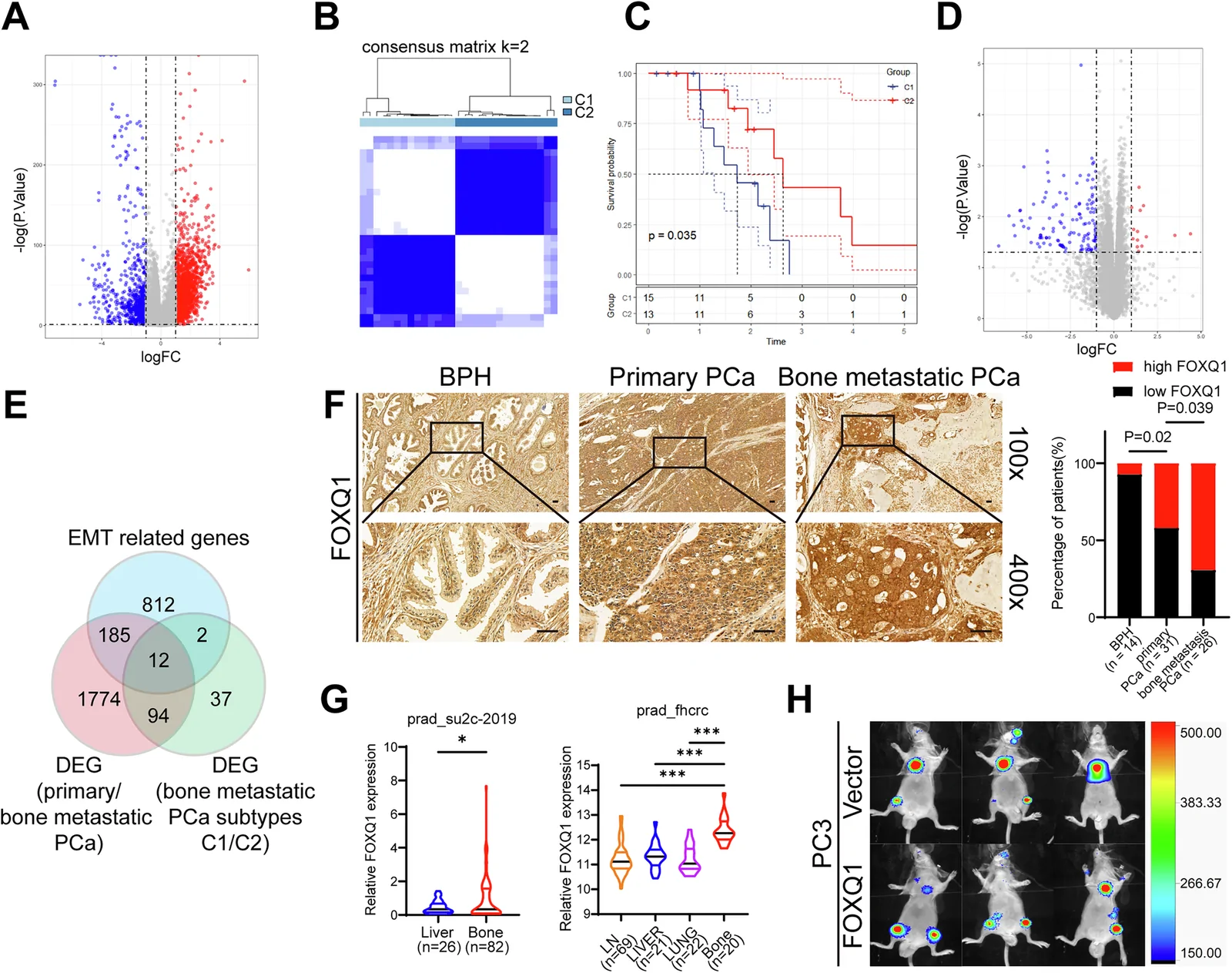
FOXQ1 is a potential key gene in PCa bone metastasis. **A** High-expression genes in bone metastatic PCa were identified based on prad_su2c_2019 and TCGA_PRAD datasets after batch correction using the ComBat algorithm. **B** Molecular classification of bone metastatic PCa was performed based on Wnt signaling pathway genes. **C** Kaplan-Meier survival analysis was conducted for the C1 and C2 subgroups. **D** Volcano plot showing differentially expressed genes in C2 compared with C1. **E** The intersecting gene FOXQ1 was identified as a potential key gene in PCa. **F** The expression of FOXQ1 in clinical samples, including BPH, primary PCa, and bone metastatic PCa, was determined by IHC. **G** Comparison of FOXQ1 expression between bone metastasis and non-bone metastasis samples using prad_su2c_2019 and prad_fhcrc datasets. **H** A left cardiac ventricle injection model was established in BALB/c-nude mice using PC3 cells with stable FOXQ1 overexpression to evaluate the effect of FOXQ1 on the bone metastatic capacity of PCa. The scale bar represents 50 μm. ***P < 0.001, **P < 0.01, *P < 0.05, ns indicates *P* ≥ 0.05.

RNA-seq data from 83 cases of bone metastatic PCa and 509 cases of primary PCa from the prad_su2c_2019 and TCGA_PRAD datasets were collected and batch-corrected. Genes significantly upregulated in bone-metastatic PCa were identified (Fig. 1A). The critical role of the Wnt pathway in bone metastasis is well established. Numerous studies have demonstrated that Wnt signaling promotes prostate cancer bone metastasis through various mechanisms, including direct induction of osteoclast differentiation and indirect modulation of the immunosuppressive microenvironment [10, 23, 24]. Molecular classification was performed using Wnt signaling pathway-related genes on bone-metastatic PCa patients with complete survival data, and the patients were divided into two subgroups: C1 and C2 (Fig. 1B). The patients in C1 group exhibited relatively poorer prognosis (Fig. 1C). Genes differentially expressed between C1 and C2 were analyzed (Fig. 1D). A total of 1011 EMT-related protein-coding genes were obtained from the dbEMT 2.0 database, and the intersection with the previously identified genes resulted in the selection of 12 potential key genes in PCa bone metastasis (Fig. 1E). Among these, FOXQ1 was highly expressed in both bone metastatic PCa and the C1 group, yet its role in PCa bone metastasis remains unclear.

Western blot and qRT-PCR analysis on FOXQ1 expression in PCa cell lines revealed that FOXQ1 expression was higher in the bone metastatic cell line PC3. FOXQ1 expression was low in C4-2 cells, but its expression was upregulated in the bone metastatic subline C4-2B cells (Fig. S1A, B). IHC analysis on the collected clinical samples revealed that FOXQ1 expression was significantly higher in PCa compared to BPH. Furthermore, FOXQ1 expression was notably elevated in bone metastatic PCa compared to primary PCa (Fig. 1F). We further compared FOXQ1 expression levels across different metastatic sites using the prad_su2c_2019 and prad_fhcrc datasets. The results showed that FOXQ1 expression was significantly higher in bone metastatic PCa than in non‑bone metastatic PCa (Fig. 1G). Moreover, intracardiac injection assays revealed that FOXQ1 upregulation markedly promoted the bone metastatic capacity of PCa cells in vivo (Fig. 1H).

### FOXQ1 promotes PCa cell proliferation and inhibits apoptosis

**Fig. 2:**
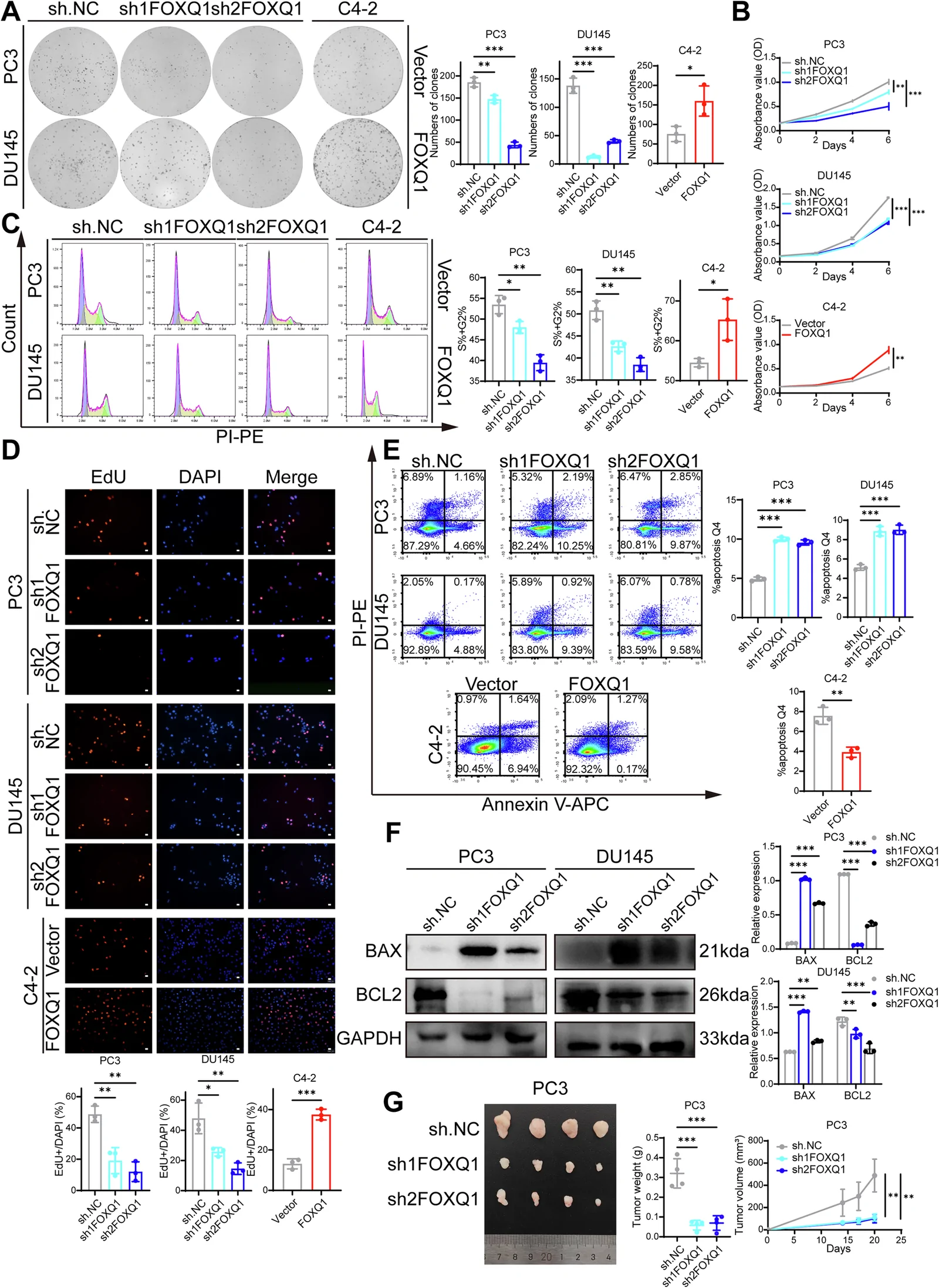
FOXQ1 promotes PCa proliferation and inhibits apoptosis. The effects of FOXQ1 knockdown on the proliferation of PC3 and DU145 cells, as well as the effects of FOXQ1 overexpression on the proliferation of C4-2 cells, were investigated using colony formation assays (**A**), CCK-8 assays (**B**), cell cycle analysis (**C**), and EdU cell proliferation assays (**D**). **E** The effects of FOXQ1 knockdown on apoptosis in PC3 and DU145 cells, and FOXQ1 overexpression on apoptosis in C4-2 cells assessed by apoptosis detection kits. **F** The expression levels of apoptosis-related proteins such as BAX and BCL2 were measured by Western blot following FOXQ1 knockdown. **G** A subcutaneous tumor model was established in BALB/c-nude mice using a stable FOXQ1 knockdown PC3 cell line to investigate the effect of FOXQ1 on tumor growth in vivo. The scale bar represents 50 μm. ***P < 0.001, **P < 0.01, *P < 0.05, ns indicates *P* ≥ 0.05.

To explore the biological function of FOXQ1 in PCa, we constructed transient interference, stable knockdown, and stable overexpression PCa cell lines using siRNA and lentivirus. Transfection efficiency was verified by qRT-PCR and Western blot (Fig. S1C–J). We examined changes in cell proliferation following FOXQ1 knockdown or overexpression using colony formation, CCK-8, cell cycle kits, and EdU proliferation assays. The results showed that FOXQ1 knockdown reduced PCa cell proliferation, while FOXQ1 overexpression significantly enhanced cell proliferation (Fig. 2A–D). Based on the TCGA database, we found that FOXQ1 expression is positively correlated with the expression of cell cycle-related genes, including CCNA1, CCND1, CCND2, CCND3, CDK2, and CDK6 (the corresponding encoded proteins are Cyclin A1, Cyclin D1, Cyclin D2, Cyclin D3, CDK2, and CDK6) in PCa tissues (Fig. S1K). Moreover, flow cytometry showed that FOXQ1 knockdown increased PCa cell apoptosis, whereas FOXQ1 overexpression suppressed apoptosis (Fig. 2E). Western blot and qRT-PCR analysis revealed that FOXQ1 expression was correlated with the expression of apoptotic proteins, with FOXQ1 knockdown resulting in upregulation of BAX and downregulation of BCL2 (Figs. 2F and S1L). A subcutaneous tumor model was established in BALB/c-nude mice, and the results showed that knockdown of FOXQ1 inhibited tumor growth in vivo (Fig. 2G).

### FOXQ1 enhances PCa cell migration

**Fig. 3:**
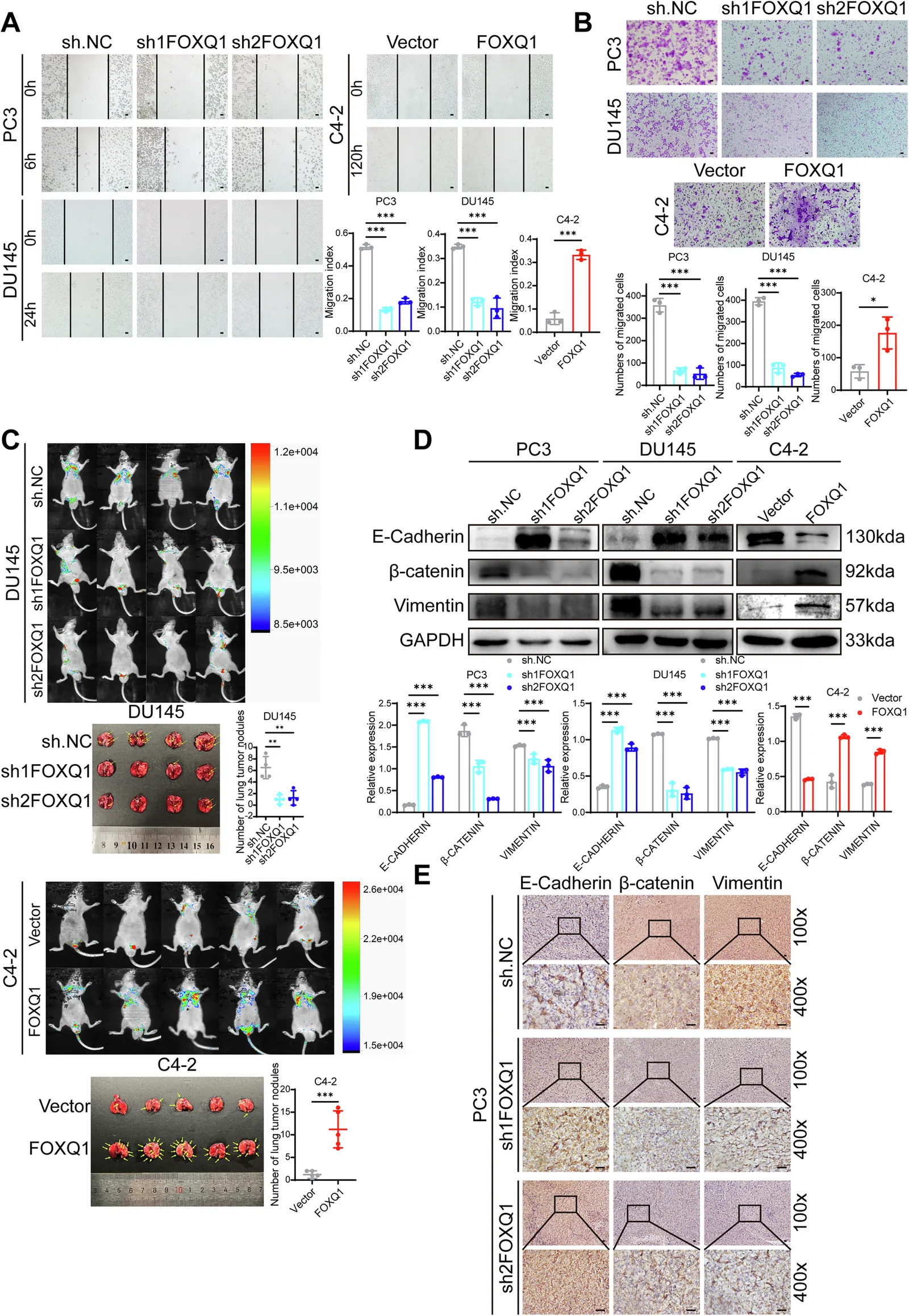
FOXQ1 promotes PCa migration. The effects of FOXQ1 knockdown on migration of PC3 and DU145 cells, and FOXQ1 overexpression on migration of C4-2 cells investigated in vitro using wound healing assays (**A**) and transwell assays (**B**). **C** A tail vein lung metastasis model was established in BALB/c-nude mice using DU145 cells with stable FOXQ1 knockdown and C4-2 cells with stable FOXQ1 overexpression to study the effect of FOXQ1 on PCa metastasis in vivo. **D** Western blot analysis examined the impact of FOXQ1 knockdown and overexpression on the expression of E-Cadherin, β-catenin, and Vimentin in PCa cells. **E** IHC staining assessed the effect of FOXQ1 knockdown on the expression of E-Cadherin, β-catenin, and Vimentin in the subcutaneous tumor model of mice. The scale bar represents 50 μm. ***P < 0.001, **P < 0.01, *P < 0.05, ns indicates *P* ≥ 0.05.

Cell migration was assessed by wound healing and transwell assays following FOXQ1 knockdown or overexpression. The results showed that FOXQ1 knockdown inhibited PCa cell migration in vitro, whereas FOXQ1 overexpression increased migration (Fig. 3A, B). Using stable knockdown and overexpression cell lines, we established a mouse tail vein metastasis model. The results revealed that FOXQ1 knockdown inhibited PCa metastasis in vivo, whereas FOXQ1 overexpression promoted metastasis (Fig. 3C). Based on the TCGA database, we found that FOXQ1 expression was positively correlated with the expression of EMT-related genes, including CTNNB1, VIM, SNAI1, and SNAI2 (the corresponding encoded proteins are β-catenin, Vimentin, Snail, and Slug) in PCa tissues (Fig. S2A). qRT-PCR showed that FOXQ1 interference increased E-Cadherin expression and decreased β-catenin, Vimentin, Snail, and Slug expression in PCa cells, with opposite results observed upon stable FOXQ1 overexpression (Fig. S2B). Western blot analysis confirmed these findings, showing that FOXQ1 knockdown upregulated E-Cadherin and downregulated Vimentin and β-catenin, with the reverse results observed upon FOXQ1 overexpression (Fig. 3D). Similar results were obtained in the subcutaneous mouse tumor tissues using IHC staining (Fig. 3E).

### FOXQ1 modulates the tumor microenvironment in PCa

**Fig. 4:**
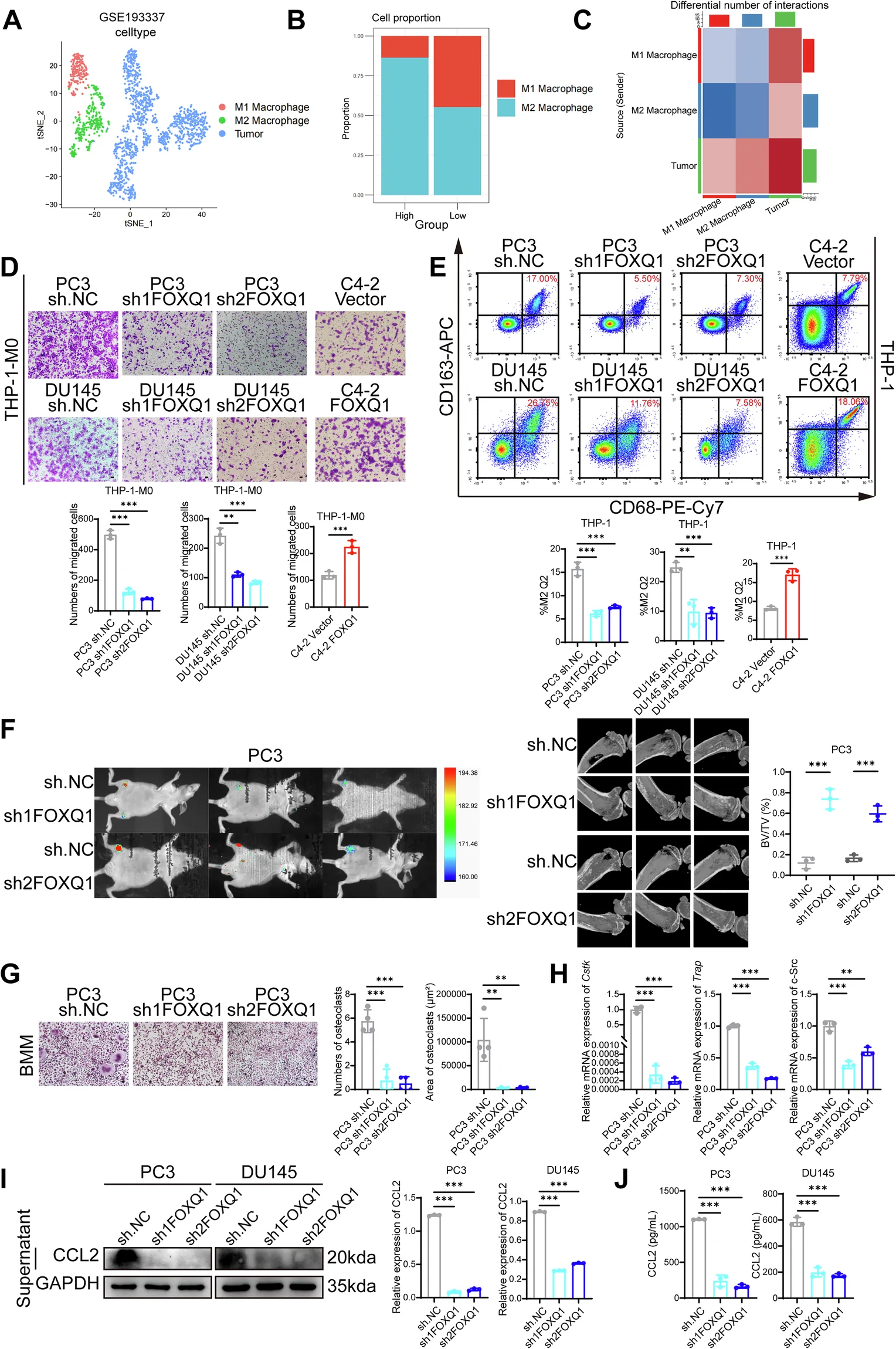
FOXQ1 regulates the tumor microenvironment in PCa. **A** Dimensionality reduction analysis of tumor and macrophage data from GSE193337. **B** The extent of M1 and M2 macrophage infiltration in FOXQ1-High and FOXQ1-Low groups was assessed. **C** The cellular communication between tumor cells and M1/M2 macrophages was investigated. **D** Transwell assays examined the effect of FOXQ1 knockdown in PC3 and DU145 cells on the recruitment of THP-1M0 (THP-1 cells induced with 100 nM PMA for 48 hours) in their supernatants, and the effect of FOXQ1 overexpression on THP-1M0 recruitment in C4-2 cell supernatants. **E** Flow cytometry evaluated the impact of FOXQ1 knockdown on THP-1 polarization induced by PC3 and DU145 cell supernatants, as well as the effect of FOXQ1 overexpression on THP-1 polarization induced by C4-2 supernatants. **F** A tibial tumor model was established in BALB/c-nude mice using PC3 cells with stable FOXQ1 knockdown to investigate the effect of FOXQ1 on PCa bone metastasis in vivo. The effects of FOXQ1 knockdown on the induction of osteoclast differentiation from BMM (isolated from mouse tibia and femur) by PC3 supernatants were assessed by TRAP staining (**G**) and qRT-PCR (**H**). The expression levels of CCL2 in the supernatants of PC3 and DU145 cells after FOXQ1 knockdown were measured by Western blot (**I**) and ELISA (**J**). The scale bar represents 50 μm. ***P < 0.001, **P < 0.01, *P < 0.05, ns indicates *P* ≥ 0.05.

Analysis using TIMER showed that FOXQ1 was associated with macrophage infiltration in the PCa tumor microenvironment (Fig. S3A). The single-cell sequencing data of PCa tissues from GSE193337 were analyzed, and the samples were grouped based on the expression levels of FOXQ1 in tumor cells (Fig. S3B–F). Subgroup analysis of tumor cells and macrophages showed that the FOXQ1-high group had a higher proportion of M2 macrophages (Fig. 4A, B). Cell communication analysis revealed that the FOXQ1-high group exhibited significantly increased communication between tumor cells and M2 macrophages (Figs. 4C and S3H, I). Based on the TCGA database, we found that FOXQ1 expression was positively correlated with the expression of key genes related to M2 macrophage, including CD163, IL-10, and MRC1 (the corresponding encoded proteins are CD163, IL-10, and CD206) in PCa tissues (Fig. S3J). Transwell assays showed that recruitment of macrophages decreased using the supernatants from FOXQ1 knockdown PCa cells, whereas supernatant from FOXQ1 overexpression PCa cells enhanced recruitment of macrophages (Figs. 4D and S3K). Co-culturing PCa cell supernatants with monocyte-derived macrophages revealed that FOXQ1 knockdown reduced the proportion of CD68^+^/CD163^+^ (CD11b^+^/CD163^+^ in murine BMMs) cells, while FOXQ1 overexpression increased the proportion (Figs. 4E and S3L).

We established a tibial tumorigenesis model using BALB/c-nude mice, and the results showed that FOXQ1 knockdown inhibited tumor formation in the tibia of PC3 cells. Additionally, micro-CT analysis revealed more severe bone destruction and lower bone density in the PC3/sh.NC group compared to the PC3/sh1FOXQ1 and PC3/sh2FOXQ1 groups (Fig. 4F). Based on the TCGA database, we found that FOXQ1 expression is positively correlated with the expression of osteoclast-related genes, including TNFSF11, ACP5, SRC, NFATc1, CTSK, and CSF1R (the corresponding encoded proteins are RANKL, TRAP, c-Src, NFATc1, CTSK, and CSF1R) in PCa tissues (Fig. S3M). Co-culture experiments showed that FOXQ1 knockdown reduced the ability of PCa cell supernatants to induce osteoclast differentiation in vitro (Fig. 4G, H).

Using TIMER and GEPIA, we found a significant positive correlation between FOXQ1 expression and CCL2 expression in PCa tissues (Fig. S3N, O). Western blot and ELISA results confirmed that FOXQ1 knockdown significantly reduced CCL2 expression in PCa cell supernatants (Fig. 4I, J). These results indicated that CCL2 could be a key factor regulated by FOXQ1 in PCa cell biological functions.

### FOXQ1 promotes PCa progression by regulating GLT8D2 transcription

**Fig. 5:**
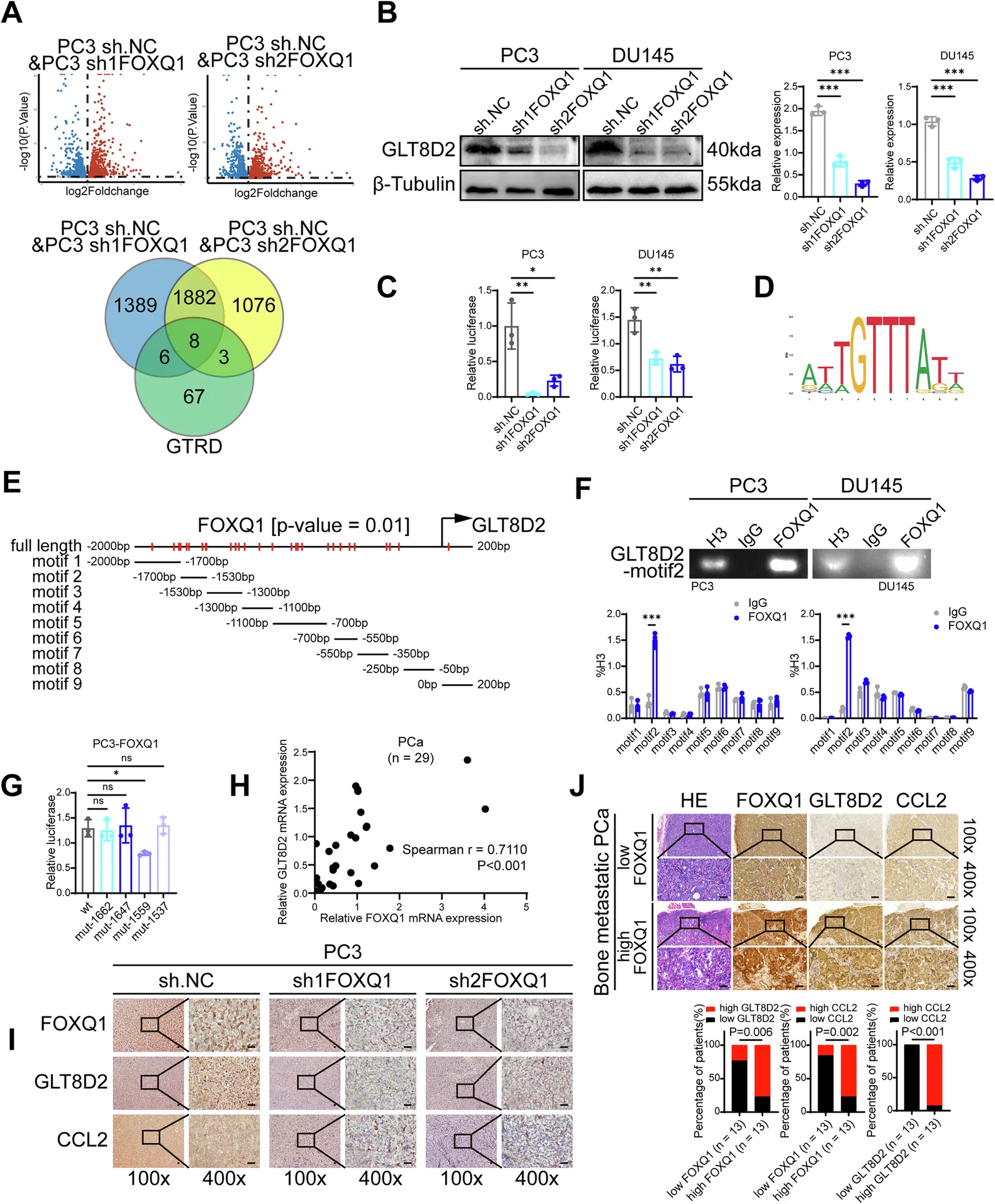
FOXQ1 promotes the transcription of GLT8D2 in PCa. **A** RNA-seq analysis identified the differentially upregulated genes in PC3/sh.NC compared to PC3/sh1FOXQ1 and PC3/sh2FOXQ1. Bioinformatic analysis predicted the downstream genes regulated by FOXQ1, and potential downstream targets of FOXQ1 in PCa. **B** Western blot analysis assessed the effect of FOXQ1 knockdown on the expression of GLT8D2 in PC3 and DU145 cells. **C** Dual-luciferase reporter assays examined the impact of FOXQ1 knockdown on GLT8D2 transcription in PC3 and DU145 cells. **D** FOXQ1 target sequence information from the JASPAR database. **E** The GeneCards platform predicted potential binding sites for FOXQ1 on the GLT8D2 promoter (–2000 bp to +200 bp), with a significance threshold of *P* < 0.01, and the promoter was divided into 9 motifs for primer design. **F** The binding of FOXQ1 to the GLT8D2 promoter was examined in the PC3 and DU145 cells. **G** Dual-luciferase reporter assays examined the transcriptional activation of GLT8D2 promoters with different mutation sites in FOXQ1-overexpressing PC3 cells. **H** qRT-PCR detected the expression correlation between FOXQ1 and GLT8D2 in 29 clinical PCa samples. IHC staining investigated the correlation of FOXQ1, GLT8D2, and CCL2 expression in a mouse subcutaneous tumor model (**I)** and clinical bone metastatic PCa samples (**J**). The scale bar represents 50 μm. ***P < 0.001, **P < 0.01, *P < 0.05, ns indicates *P* ≥ 0.05.

Given the transcription factor properties of FOXQ1, we used RNA-seq to analyze genes differentially expressed in PC3/sh.NC versus PC3/sh1FOXQ1 and PC3/sh2FOXQ1 cells. The downstream genes targeted by FOXQ1 for binding and transcriptional promotion were predicted using GTRD. The intersection of these results identified GLT8D2 as a high-confidence target gene of FOXQ1, while CCL2 is less likely to be directly regulated by FOXQ1 (Fig. 5A). Western blot analysis confirmed that FOXQ1 knockdown reduced GLT8D2 expression in PCa cells (Fig. 5B). Dual-luciferase reporter assays showed that FOXQ1 knockdown significantly reduced GLT8D2 transcription levels (Fig. 5C). Based on FOXQ1 binding sites predicted by the JASPAR database (Fig. 5D), GeneCards was utilized to predict potential FOXQ1 binding sites in the GLT8D2 promoter (–2000bp ~ +200 bp) with a *P*-value < 0.01, leading to the identification of nine potential FOXQ1 binding motifs (Fig. 5E). ChIP assays confirmed that FOXQ1 binds to the GLT8D2-motif2 region (Fig. 5F). Using FOXQ1-overexpressing PC3 cells, we examined the transcriptional activation of GLT8D2 promoters with mutations at different predicted binding sites. The results identified the –1559bp region as a potential transcriptional binding site for FOXQ1 (Fig. 5G). qRT-PCR analysis of FOXQ1 and GLT8D2 expression in clinical PCa tissues revealed a significant positive correlation between the two genes (Fig. 5H).

Analysis using TIMER and GEPIA further revealed a positive correlation between FOXQ1, GLT8D2 and CCL2 expression (Fig. S4A–C). IHC performed on both the mouse subcutaneous tumor tissues and clinical specimens revealed similar results (Fig. 5I, J). Further analysis using TIMER and GEPIA revealed a positive correlation between GLT8D2 expression and macrophage infiltration in the tumor microenvironment of PCa (Fig. S4D). Additionally, GLT8D2 expression in PCa tissues was positively correlated with the expression of EMT-related genes, including CTNNB1, VIM, SNAI1, and SNAI2 (encoded proteins: β-catenin, Vimentin, Snail, Slug), M2 macrophage-associated genes such as CD163, IL10, and MRC1 (encoded proteins: CD163, IL-10, CD206), and osteoclast-related genes such as TNFSF11, ACP5, SRC, NFATc1, CTSK, and CSF1R (encoded proteins: RANKL, TRAP, c-Src, NFATc1, CTSK, CSF1R) (Fig. S4E–G).

**Fig. 6:**
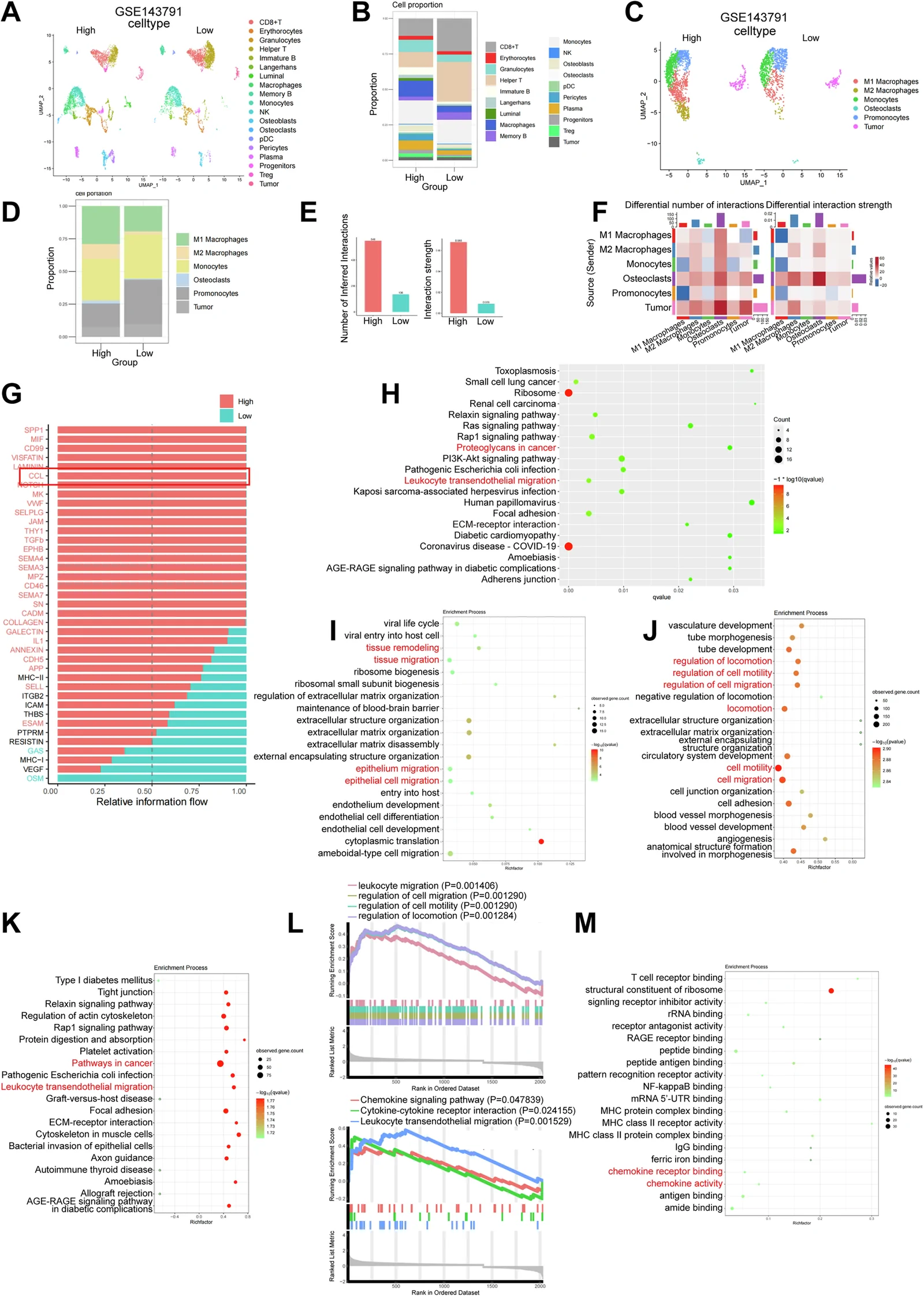
The impact of GLT8D2 on bone metastatic PCa analyzed using single-cell sequencing results. **A** Analysis of the single-cell sequencing data from the GSE143791 dataset in bone metastatic PCa, with samples grouped based on the expression levels of GLT8D2 in the tumor. **B** The cell composition was identified using stacked bar charts. **C** Identification of monocytes, macrophages, osteoclasts, and tumor cells, followed by dimensionality reduction. **D** The cell composition identified using stacked bar charts. **E** The intensity of cell interactions in GLT8D2-high versus GLT8D2-low samples. **F** The differences of cell communication between various cell components in GLT8D2-high versus GLT8D2-low samples. **G** The intensity of cell communication pathways in GLT8D2-high versus GLT8D2-low samples. **H** KEGG pathway analysis of upregulated genes in tumor cells from the GLT8D2-high group relative to the GLT8D2-low group. **I** GO_BP analysis of the biological processes enriched in upregulated genes from tumor cells in the GLT8D2-high group relative to the GLT8D2-low group. **J** GSEA_GO_MF analysis of the molecular functions enriched in differentially expressed genes from tumor cells in the GLT8D2-high group relative to the GLT8D2-low group. **K** GSEA_KEGG analysis of enriched pathways in differentially expressed genes from tumor cells in the GLT8D2-high group relative to the GLT8D2-low group. **L** Peak plots illustrating the enrichment of pathways in differentially expressed genes from tumor cells in the GLT8D2-high group relative to the GLT8D2-low group, based on GSEA_GO_BP and GSEA_KEGG analysis. **M** GO_MF analysis of the biological processes enriched in upregulated genes in macrophages from the GLT8D2-high group relative to the GLT8D2-low group.

Analysis of GSE143791 single-cell sequencing data from bone metastatic PCa revealed that the GLT8D2-high group had higher macrophage infiltration (Fig. 6A, B). Further analysis identified differentially expressed genes in macrophages, osteoclasts, tumor cells, and other immune cell subsets (Figs. 6C and S5D). Pseudotime analysis was utilized to confirm the accuracy of the cell subset grouping (Fig. S5E–J). The GLT8D2-high group exhibited higher levels of M2 macrophage and osteoclast infiltration, as well as enhanced tumor-to-tumor, tumor-to-M2 macrophage, and tumor-to-osteoclast communication, with the CCL signaling pathway significantly upregulated (Figs. 6D–G and S5K, L). KEGG enrichment analysis showed that genes upregulated in the GLT8D2-high group were significantly enriched in proteoglycans in cancer and leukocyte transendothelial migration (Fig. 6H). GO enrichment analysis revealed that genes highly expressed in tumor cells of the GLT8D2-high group were enriched in cellular components like the endoplasmic reticulum and endoplasmic reticulum lumen (Fig. S5M), and were associated with biological processes such as tissue remodeling and epithelial cell migration (Fig. 6I). GSEA enrichment analysis yielded similar results and revealed that differentially expressed genes in the GLT8D2-high group were significantly associated with cytokine interactions and chemokine signaling pathways (Fig. 6J–L). Additionally, by screening for upregulated genes within the macrophage subpopulation in the GLT8D2-high group and performing GO enrichment analysis, it was found that those genes were enriched in chemokine receptor binding and chemokine activity (Fig. 6M).

**Fig. 7:**
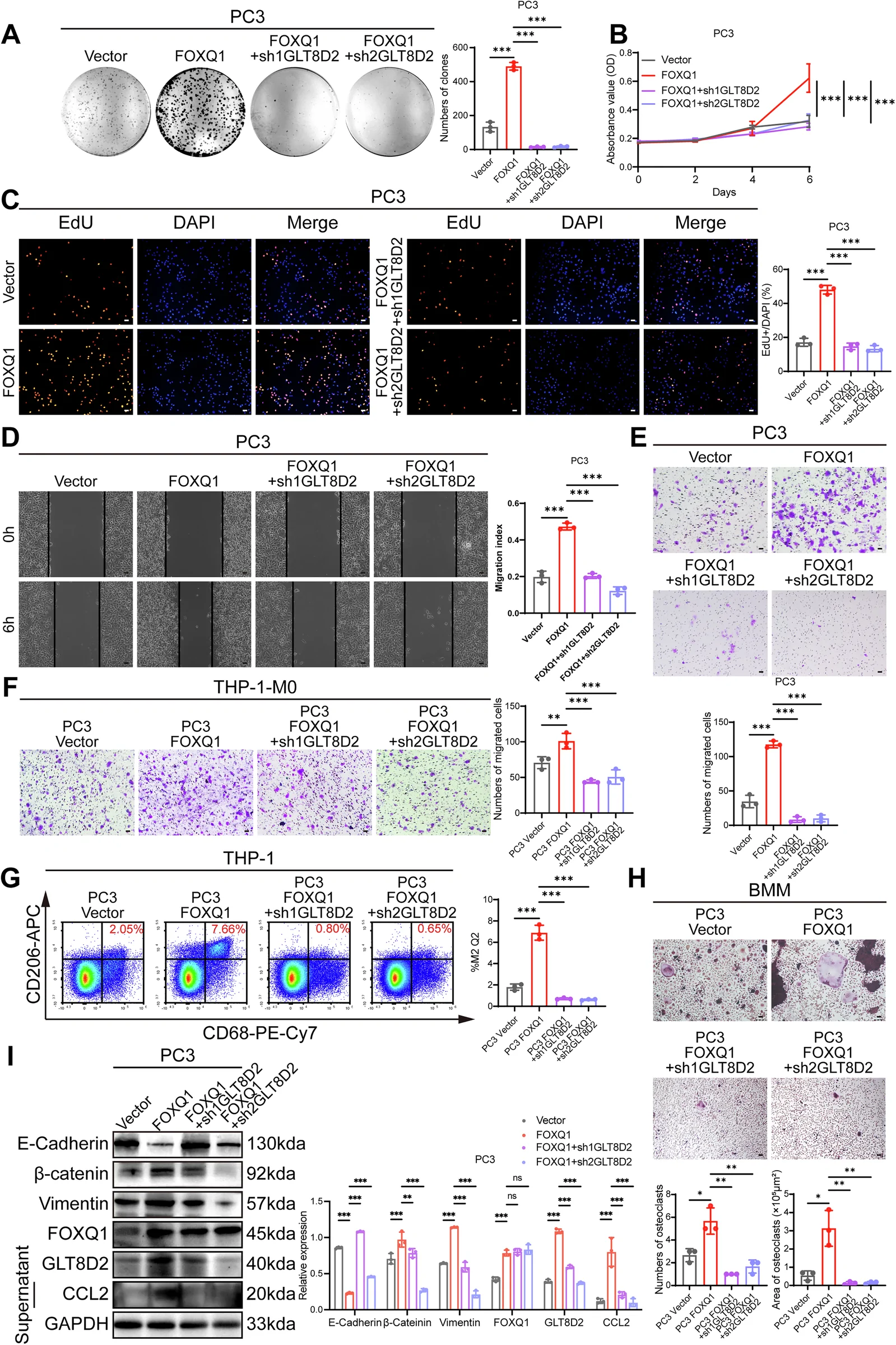
FOXQ1 regulates the progression of PCa through GLT8D2. The effect of GLT8D2 knockdown in PCa on FOXQ1-induced proliferation of PC3 cells investigated using colony formation assays (**A**), CCK-8 assays (**B**), and EdU proliferation assays (**C**). The impact of GLT8D2 knockdown in PC3 cells on FOXQ1-induced migration evaluated through wound healing assays (**D**) and transwell assays (**E**). **F** The effect of GLT8D2 knockdown in PC3 cells on FOXQ1-induced recruitment of THP-1 M0 cells examined using transwell assays. **G** Flow cytometry analysis assessed the impact of GLT8D2 knockdown in PC3 cells on FOXQ1-induced polarization of THP-1 cells to M2 macrophages. **H** TRAP staining employed to examine the effect of GLT8D2 knockdown in PC3 cells on FOXQ1-induced differentiation of BMMs into osteoclasts. **I** Western blot analysis was conducted to study the effect of GLT8D2 knockdown in PC3 cells on the expression of proteins regulated by FOXQ1 overexpression, including E-Cadherin, β-catenin, Vimentin, FOXQ1, GLT8D2, and CCL2 in the supernatant. The scale bar represents 50 μm. ***P < 0.001, **P < 0.01, *P < 0.05, ns indicates *P* ≥ 0.05.

Western blot was used to assess the knockdown efficiency of GLT8D2 by the constructed lentivirus (Fig. S5N). In vitro studies were used to investigate the effect of GLT8D2 knockdown on the ability of FOXQ1 to promote PCa progression. Colony formation, CCK-8, and EdU proliferation assays demonstrated that GLT8D2 knockdown reversed the proliferative effect of FOXQ1 on PCa cells (Fig. 7A–C). Wound healing and transwell assays showed that GLT8D2 knockdown reversed the effect of FOXQ1 in promoting PCa cell migration (Fig. 7D, E). Co-culture experiments revealed that GLT8D2 knockdown reversed the role of FOXQ1 in recruiting tumor-associated macrophages and promoting M2 polarization (Fig. 7F, G), and reversed FOXQ1’s ability to promote osteoclast differentiation (Fig. 7H). Western blot analysis showed that GLT8D2 knockdown reversed FOXQ1-induced downregulation of E-cadherin and upregulation of β-catenin, Vimentin, and CCL2, while FOXQ1 expression was not significantly affected by GLT8D2 knockdown (Fig. 7I). Overall, FOXQ1 promotes PCa bone metastasis by activating GLT8D2 transcriptional regulation.

### GLT8D2 enhances the N-glycosylation of CCL2

**Fig. 8:**
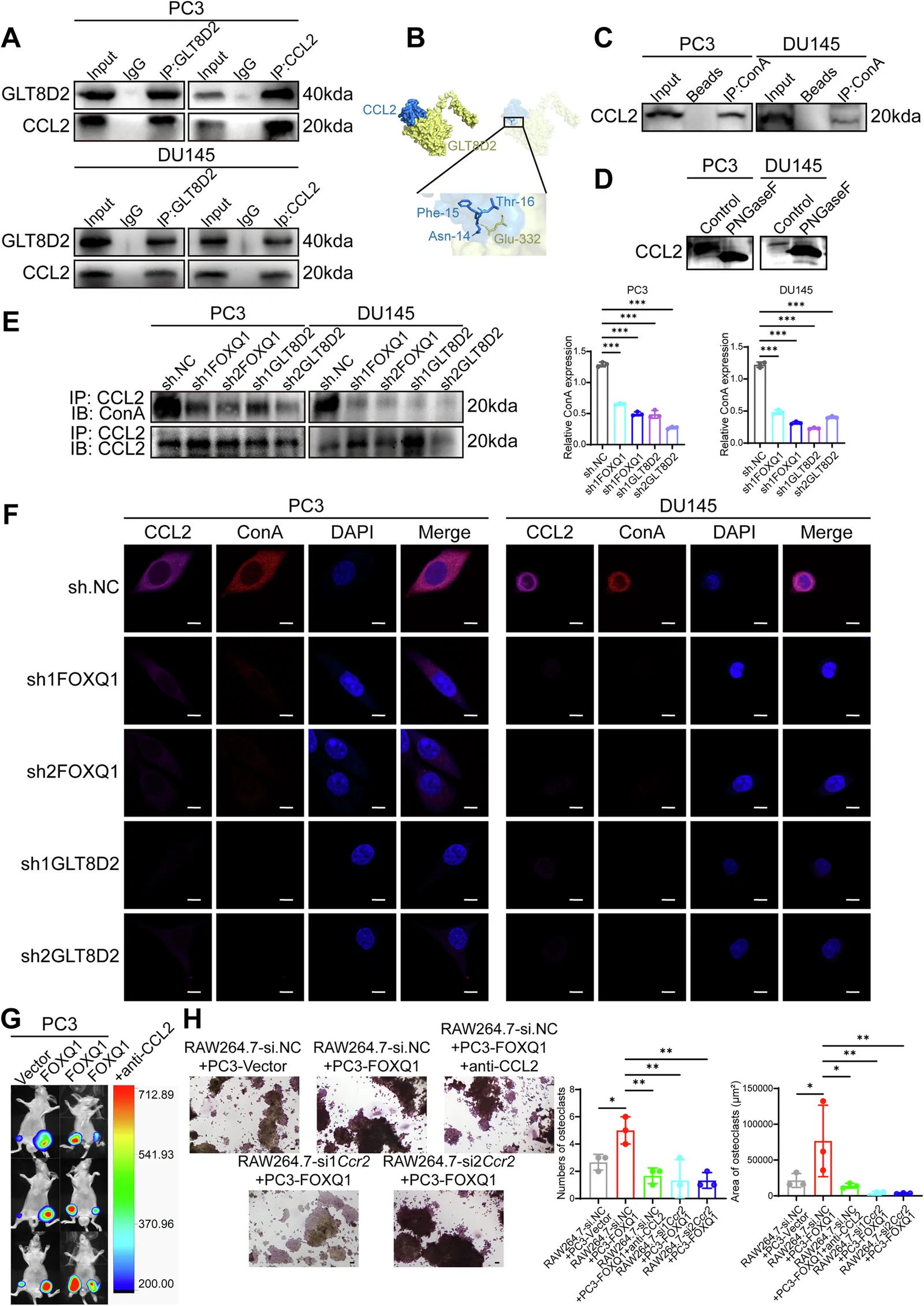
FOXQ1 enhances the N-glycosylation of CCL2 through GLT8D2. **A** Co-IP assessed the interaction between GLT8D2 and CCL2 in PC3 and DU145 cells. **B** A molecular docking prediction model was constructed to predict the binding sites of GLT8D2 with CCL2. **C** ConA-pulldown assay detected the N-glycosylation modification of CCL2 in PC3 and DU145 cells. **D** PNGaseF treatment cleaved N-glycans, and Western blot analysis monitored the changes in the molecular weight of CCL2. **E** Co-IP was used to obtain CCL2 from PC3 and DU145 cells, followed by Lectin blot and Western blot analysis to detect the N-glycosylation of CCL2 and its modulation by FOXQ1 and GLT8D2 knockdown. **F** Confocal immunofluorescence imaging examined the co-localization of CCL2 with N-glycosylation in PC3 and DU145 cells, as well as the effects of FOXQ1 or GLT8D2 knockdown on their expression. **G** Local administration of CCL2 neutralizing antibody significantly reversed the promoting effect of FOXQ1 overexpression on PCa bone metastasis in a tibial injection model. **H** Both the CCL2 neutralizing antibody and the knockdown of *Ccr2* in RAW264.7 cells effectively abrogated the promoting effect of FOXQ1 overexpression on osteoclast differentiation in a co-culture system. The scale bar represents 10 μm. ***P < 0.001, **P < 0.01, *P < 0.05, ns indicates *P* ≥ 0.05.

We found that GLT8D2 expression correlated with CCL2 in PCa, and that GLT8D2 knockdown reversed the FOXQ1-induced upregulation of CCL2 expression. Co-IP assays confirmed an interaction between GLT8D2 and CCL2 in PCa cells (Fig. 8A). Molecular docking model predictions indicated that the N-glycosylation site Asn-14 of CCL2 is a high-confidence binding site for GLT8D2 (Fig. 8B). ConA-pulldown assays showed N-glycosylation modification of CCL2 in PCa cells (Fig. 8C). Total proteins from PCa cells were collected and treated with PNGaseF to cleave the N-glycans. Western blot analysis revealed a reduction in the molecular weight of CCL2 protein (Fig. 8D). Lectin blotting assay showed that FOXQ1 or GLT8D2 knockdown significantly decreased CCL2 N-glycosylation (Fig. 8E). Immunofluorescence microscopy showed colocalization of ConA-labeled N-glycosylation sites and CCL2 in PCa cells. Moreover, the levels of N-glycosylation and CCL2 expression were significantly reduced after FOXQ1 or GLT8D2 knockdown (Fig. 8F). These results suggest that GLT8D2 promotes CCL2 expression by enhancing its N-glycosylation.

Functionally, local administration of a CCL2 neutralizing antibody in a tibial injection model reversed the promoting effect on bone metastasis induced by PCa cells with stable FOXQ1 overexpression (Fig. 8G). Consistently, in a co-culture system, treatment with CCL2 neutralizing antibody or the knockdown of *Ccr2* in RAW264.7 cells abrogated the enhancement of osteoclast differentiation induced by FOXQ1-overexpressing PCa cells (Figs. 8H and S5O).

## Discussion

Bone metastatic PCa remains a significant challenge in clinical treatment. It lacks satisfactory therapeutic options and is associated with a poor prognosis for patients. In this study, we identified FOXQ1 as a potential key gene involved in PCa bone metastasis through screening public databases. Although FOXQ1 has been implicated in playing critical roles in various types of cancers, its role in PCa remains unexplored, and its impact on PCa bone metastasis and molecular mechanisms has not been studied. To investigate the effects of abnormal FOXQ1 expression on the pathogenesis of PCa, we conducted a series of bioinformatics analyses and in vitro and in vivo functional studies. Our results indicate that FOXQ1 promotes PCa cell proliferation, migration, and inhibits apoptosis. Additionally, the aberrant upregulation of FOXQ1 in PCa enhances macrophage recruitment, M2 polarization, and osteoclast differentiation in the tumor microenvironment.

CCL2, also known as MCP-1 (Monocyte Chemoattractant Protein-1), is one of the five monocyte chemoattractants identified in the CC chemokine family [25]. CCL2 has been shown to recruit macrophages and induce M2 polarization [26]. CCL2 is secreted by various cells, including tumor cells, endothelial cells, fibroblasts, and activated monocytes. The main receptor for CCL2, Chemokine Receptor 2 (CCR2), is expressed in several cell types, including dendritic cells, endothelial cells, monocytes, and tumor cells [27]. CCL2 can influence tumor progression through various pathways, and it has been found to regulate tumor growth and metastasis in various types of cancers [28, 29]. Previous studies suggest that CCL2 promotes PCa bone metastasis by regulating osteoclast growth and differentiation [30–32]. The biological functions of CCL2 are highly similar to those of FOXQ1. Interestingly, FOXQ1 has been shown to upregulate CCL2 expression through Twist1 and Versicanv1, thereby promoting the progression of colorectal and hepatocellular carcinoma [33, 34]. In our study, we found that FOXQ1 enhances CCL2 secretion in PCa cells. However, based on sequencing results and public database information, the possibility of FOXQ1 directly regulating CCL2 transcription in PCa is low, which is consistent with previous findings. Further screening led us to identify GLT8D2, a high-confidence downstream target gene of FOXQ1.

GLT8D2 was identified as a novel glycosyltransferase in 2013 [35]. Research on the biological function of GLT8D2 and its role in disease is still in the early stages. Existing studies indicate that GLT8D2 is associated with pulmonary arterial hypertension [36], adenomyosis [37], osteoporosis [38], and other conditions. In malignancies, bioinformatics analysis has linked GLT8D2 to poor prognosis in bladder urothelial carcinoma [39] and gastric cancer [40–42]. The molecular mechanisms of GLT8D2 remain unclear. Some studies suggested that it induces non-alcoholic fatty liver disease by downregulating MTP expression [43], and may activate the FGFR/PI3K/AKT pathway to induce CDDP resistance in ovarian cancer [44]. However, the specific glycosylation modification pathways regulated by GLT8D2 have not been reported. In our study, we found that FOXQ1 promotes PCa progression via activating GLT8D2 to enhance PCa cell proliferation, migration, and macrophage recruitment, M2 polarization, and osteoclast differentiation in the tumor microenvironment. Bioinformatics analysis indicates that high GLT8D2 expression in bone metastatic PCa cells facilitates cell communication between tumor-tumor, tumor-M2 macrophage, and tumor-osteoclast, and is associated with tumor cell migration, leukocyte migration, and chemokine signaling pathways. Mechanistic studies revealed that GLT8D2 is transcriptionally regulated by FOXQ1, leading to increased CCL2 expression in PCa. Further investigations showed that GLT8D2 influences CCL2 expression by promoting its N-glycosylation modification in PCa cells. Notably, glycosylation is a critical post-translational modification of CCL2 [45], with the protein structure containing both an N-glycosylation site and an O-glycosylation site. The N-glycosylation modification significantly enhances CCL2 protein stability and secretion efficiency, which is essential for CCL2 to perform its biological functions effectively [46]. Our study provides important insights into the function of GLT8D2 in glycosylation modification. Future studies are warranted to further define the substrate specificity and catalytic mechanism of GLT8D2.

In recent years, significant progress has been made in targeting transcription factors, particularly with the use of enzalutamide targeting the androgen receptor and tamoxifen targeting the estrogen receptor, both of which have been widely applied in the treatment of PCa and breast cancer [47, 48]. The regulatory role of FOXQ1 in tumors has attracted considerable attention. Studies have shown that in hepatocellular carcinoma, JNK1 upregulates FOXQ1 transcriptional activity through phosphorylation at Ser-248 [49], while resveratrol directly activates SIRT1 in colorectal cancer to suppress FOXQ1 expression [50]. These findings suggest that FOXQ1 may serve as a potential prognostic marker or a predictor of bone metastasis in prostate cancer. However, its therapeutic applicability remains challenging due to the intrinsic difficulties of targeting transcription factors, and its potential adverse effects in bone metastatic PCa require further investigation. Additionally, therapies targeting CCL2 and its main receptor CCR2 have been the focus of research, with drugs such as Bindarit and Carlunmab targeting CCL2, and GMME1 and MLN1202 targeting CCR2, some of which have entered clinical trials. However, the sustained inhibitory effects of these drugs on CCL2/CCR2 and their potential systemic impacts remain unresolved [27]. Our study proposes a mechanism in which GLT8D2 regulates the upregulation of CCL2 N-glycosylation in PCa cells, promoting bone metastasis. Targeted inhibition of N-glycosylation or specific removal of the N-glycans on CCL2 might represent a novel approach to suppress CCL2 in the tumor microenvironment, although its feasibility and specificity require further investigation.

The initial gene screening in this study integrated multiple public datasets, which exhibit potential heterogeneity in sample acquisition, processing, disease context, tumor purity, and microenvironmental composition. Such heterogeneity cannot be fully eliminated by batch correction and may influence the screening results. Nonetheless, we validated the central role of the FOXQ1/GLT8D2/CCL2 axis through multi-level functional experiments. Furthermore, the molecular subtyping based on Wnt pathway-related genes was primarily intended to identify prognostic patient groups and narrow the candidate gene screen, rather than to presuppose Wnt-driven biology. The specific relationship between FOXQ1 and the Wnt pathway was not systematically investigated here and warrants future study. Prospective cohorts with uniformly processed, paired primary and bone metastatic samples will further consolidate our findings.

## Conclusions

**Fig. 9:**
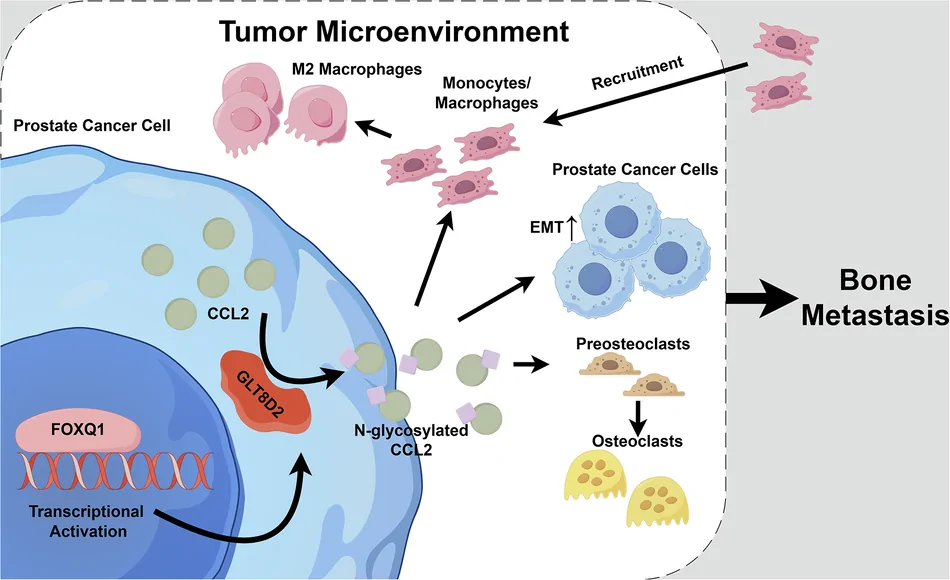
Illustration showing the mechanism of how FOXQ1 activates the transcription of GLT8D2, leading to the upregulation of CCL2 N-glycosylation modification, which promotes prostate cancer bone metastasis (by Figdraw).

In summary, our research demonstrates that FOXQ1 indirectly enhances CCL2 N-glycosylation modification by activating GLT8D2 transcription, thereby upregulating CCL2 expression in PCa cells, ultimately promoting PCa bone metastasis. Our findings on the FOXQ1-GLT8D2-CCL2 axis provide new insights into PCa bone metastasis and offer promising biomarkers for risk prediction and precision treatment in bone metastatic PCa (Fig. 9).

## Supplementary information

Western blot_Agarose Gel Electrophoresis_Lecin blot_Raw data

supplementary

RNA-seq NC&SH2

RNA-seq NC&SH3

## Data Availability

The RNA-seq data generated in this study are available in the supplementary materials. The gene expression publicly available data used in this study are available in GEO under the accession codes GSE193337 and GSE143791. Data relevant to prad_su2c_2019 and prad_fhcrc were obtained from cBioPortal.
