# Supplementary material for: FOXQ1 activates GLT8D2 to enhance CCL2 N-glycosylation and promote prostate cancer bone metastasis
Source: Cell Death Dis. 2026 Jul 1;17(1):817. doi: 10.1038/s41419-026-09046-9 (PMC13590482; doi:10.1038/s41419-026-09046-9)

Fig. 2F

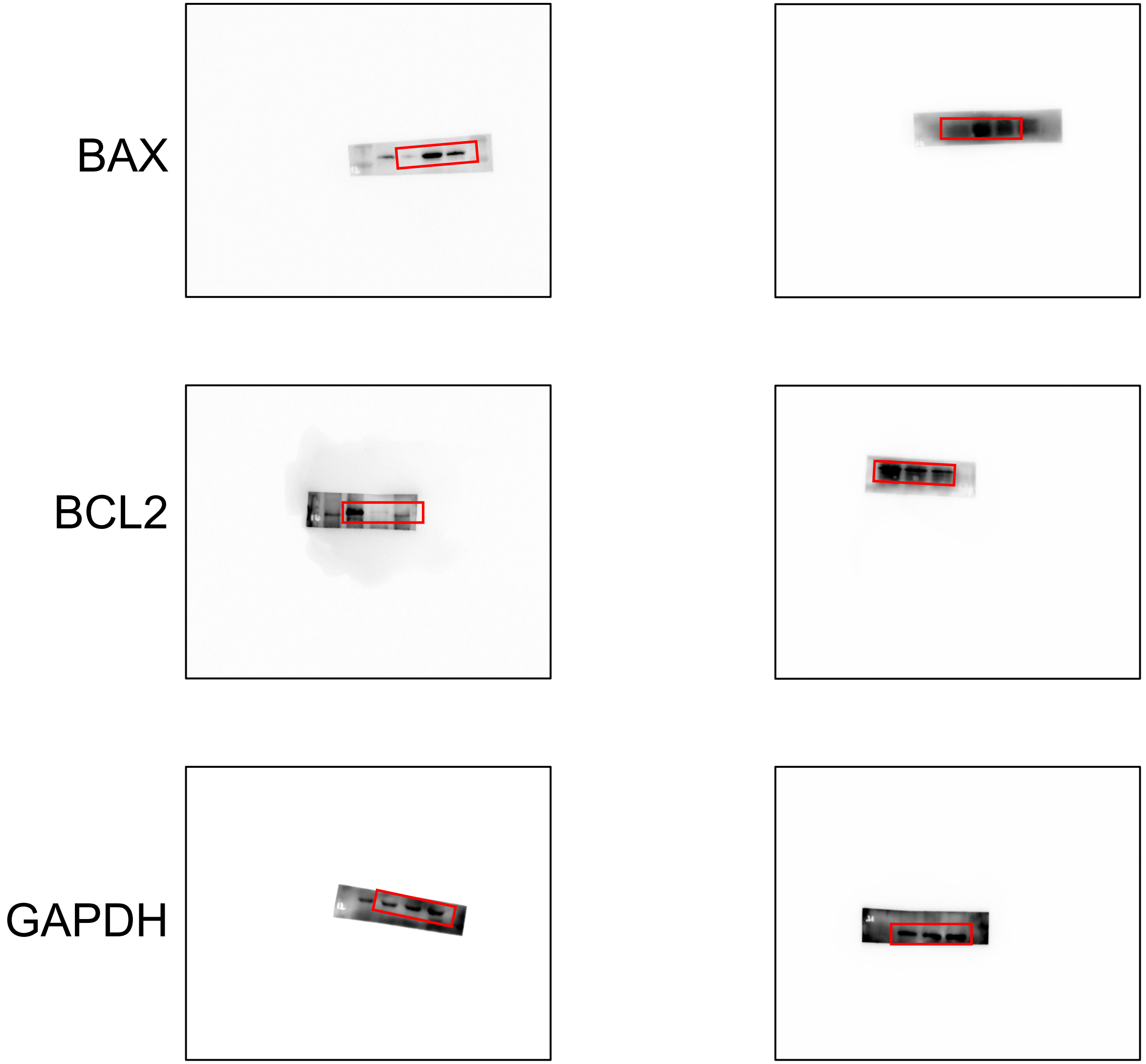

Fig. 3D

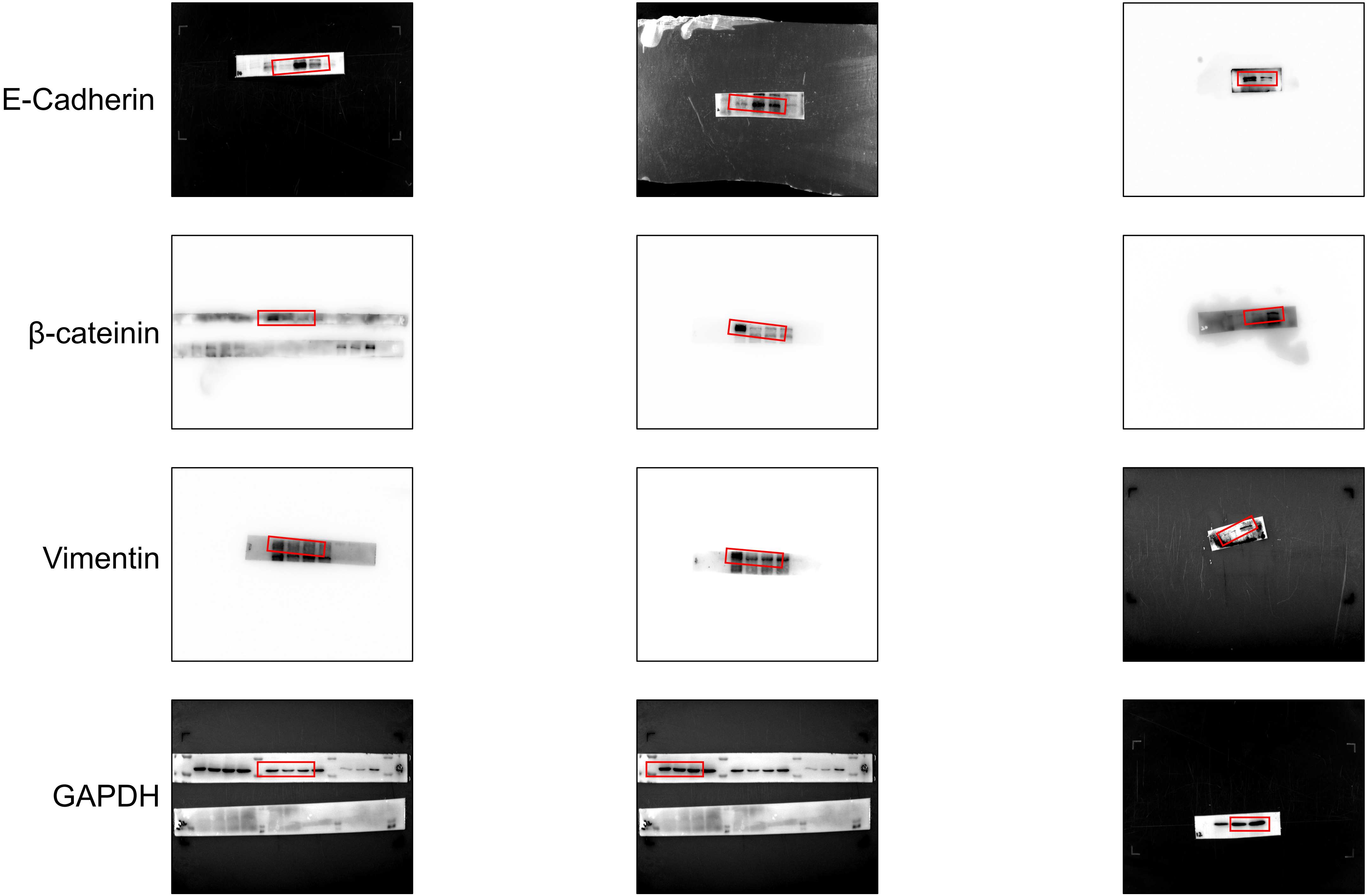

Fig. 4I

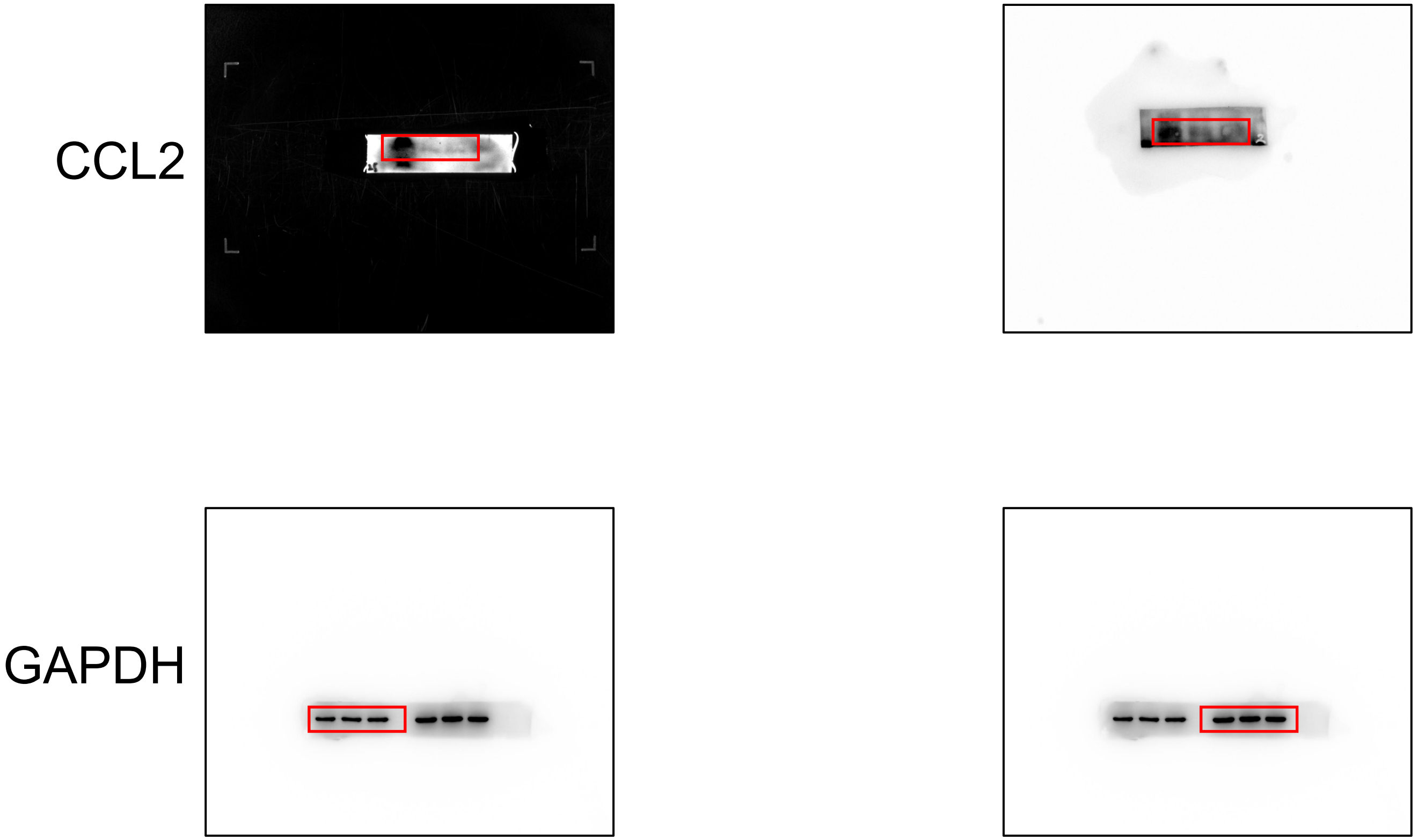

Fig. 5B

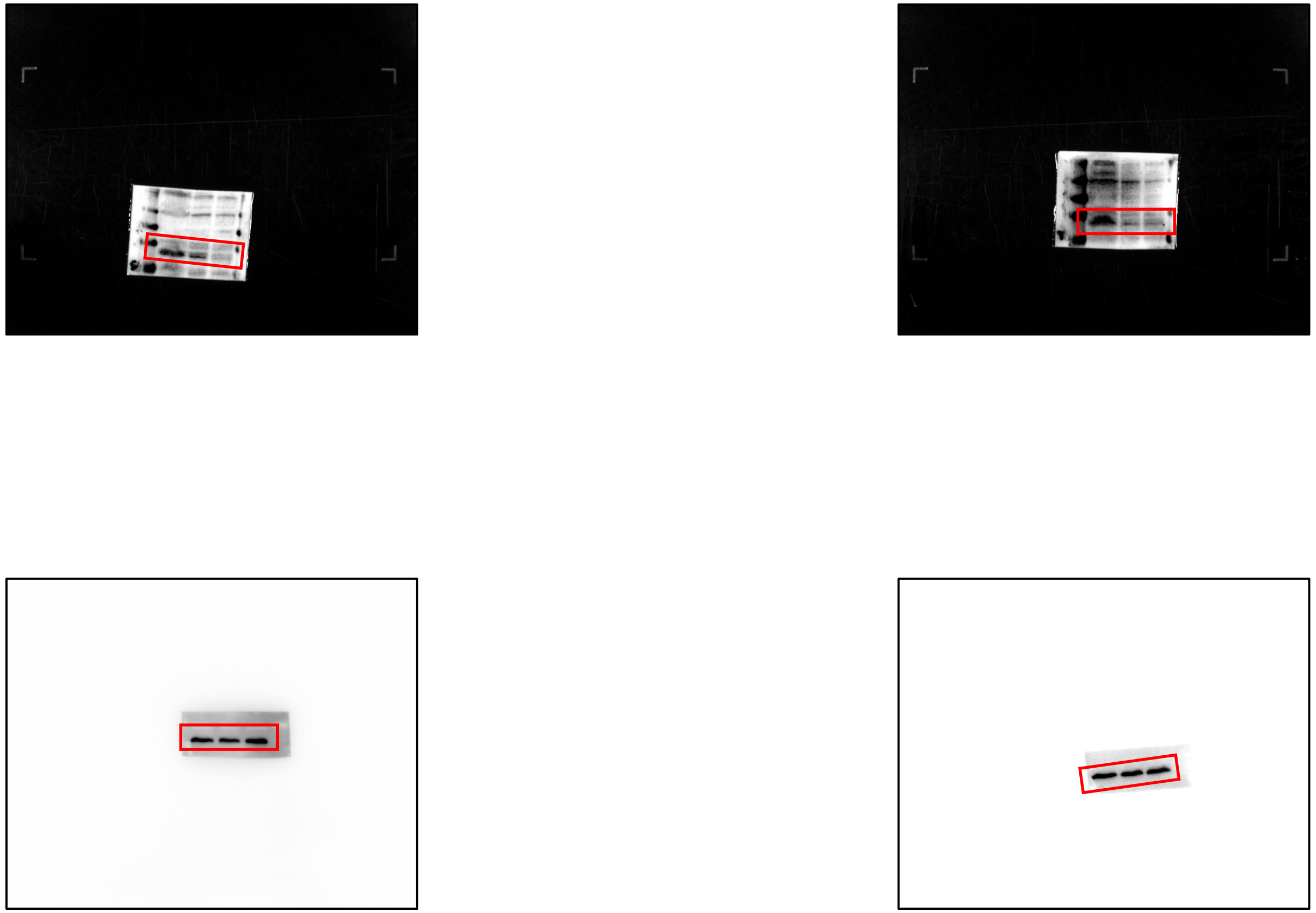

GLT8D2

β-Tubulin

Fig. 5F

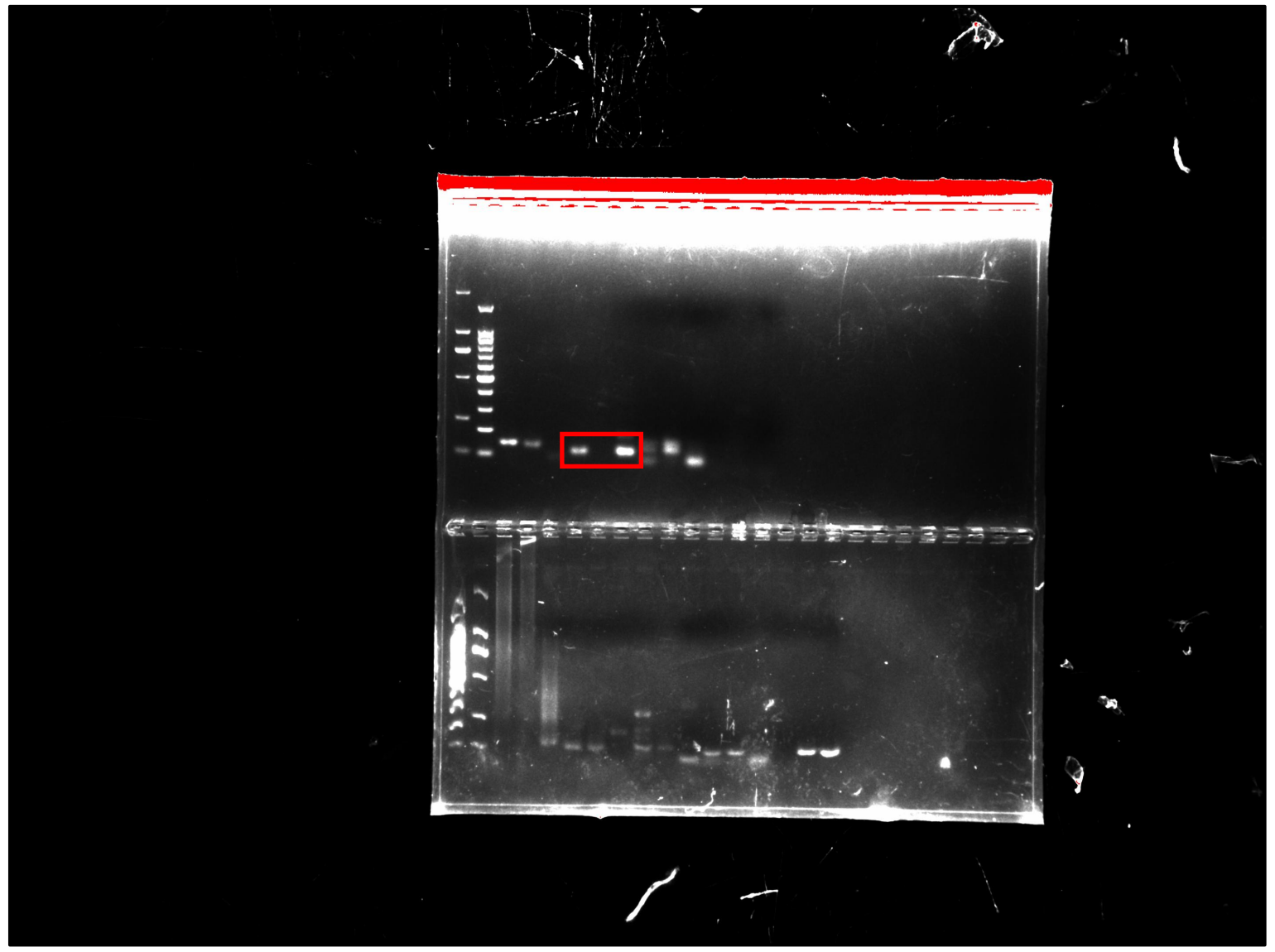

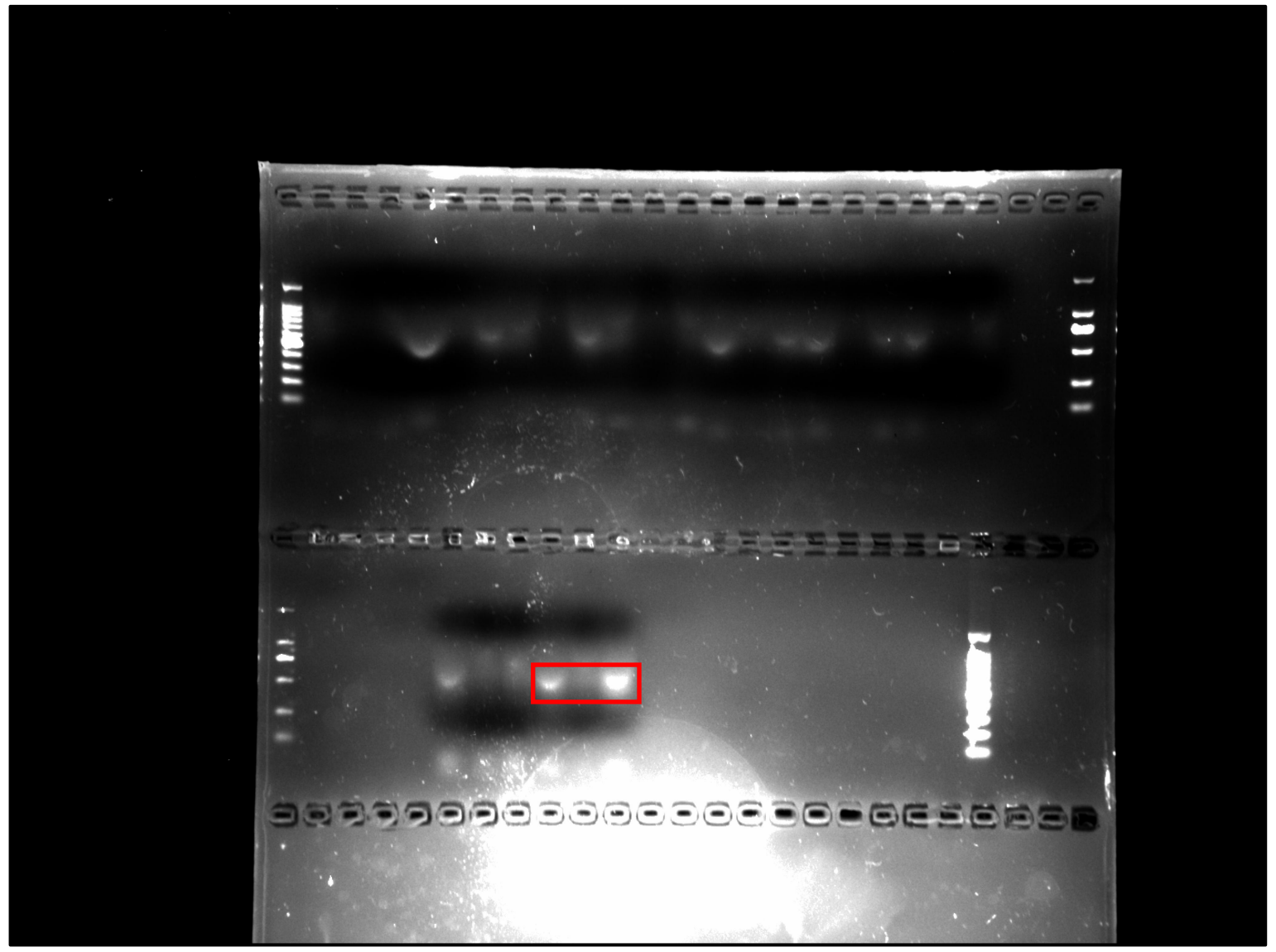

Fig. 7I

E-Cadherin

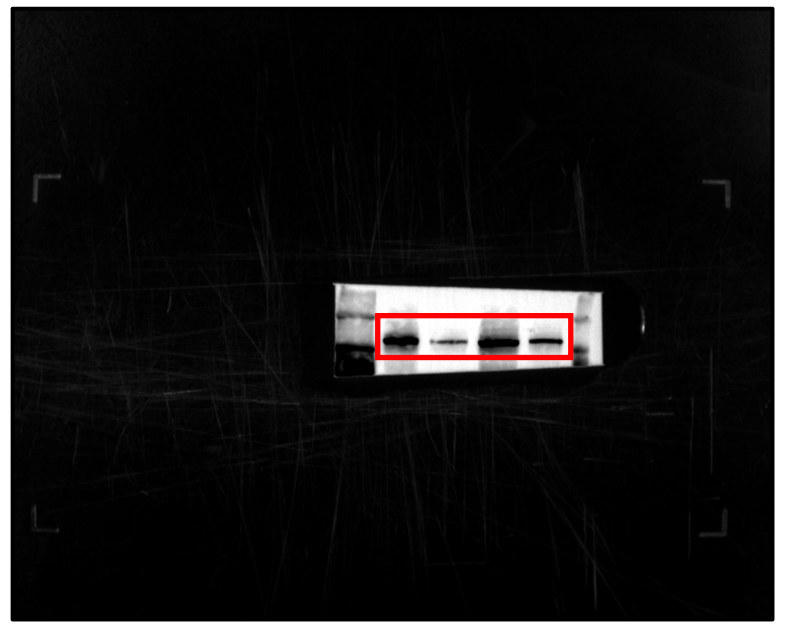

β-cateinin

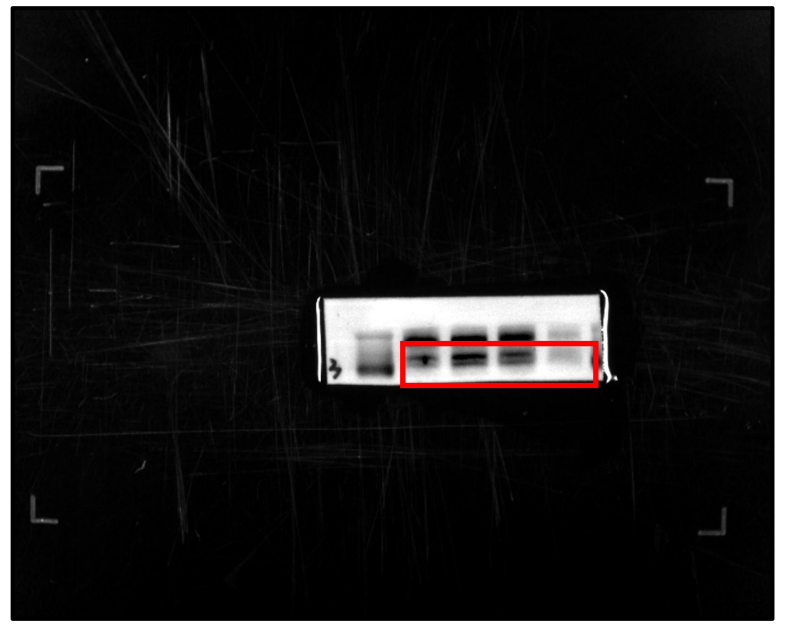

Vimentin

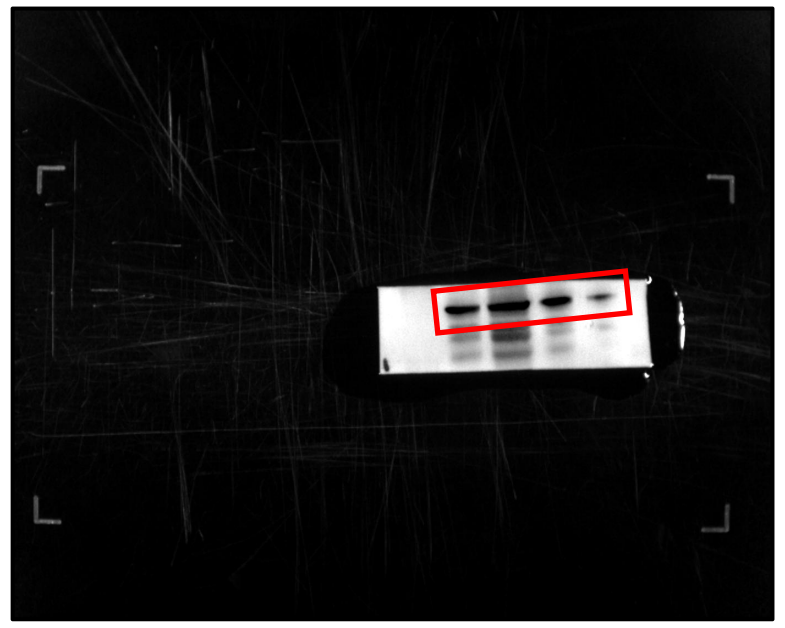

FOXQ1

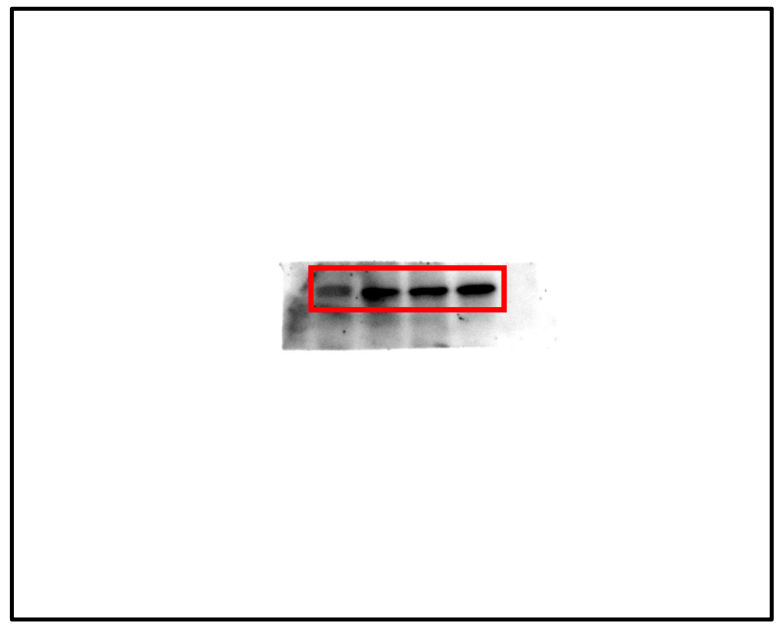

GLT8D2

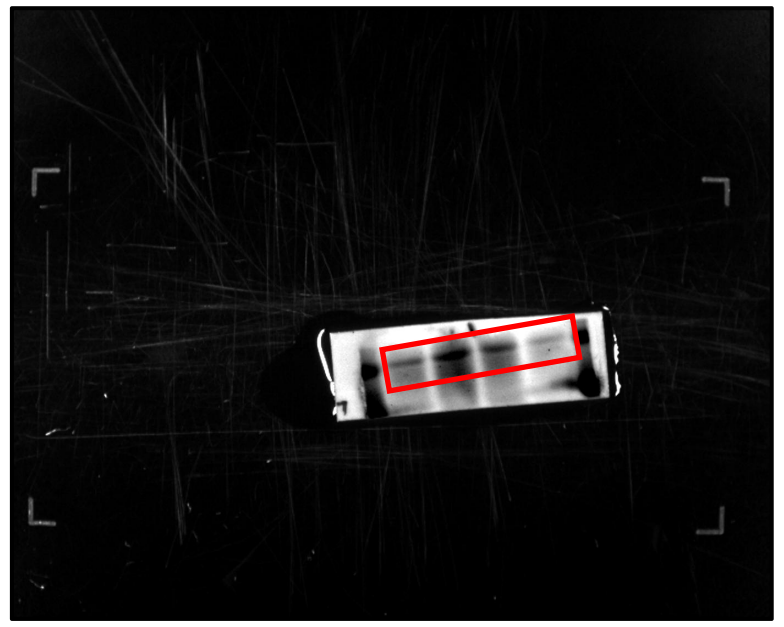

CCL2

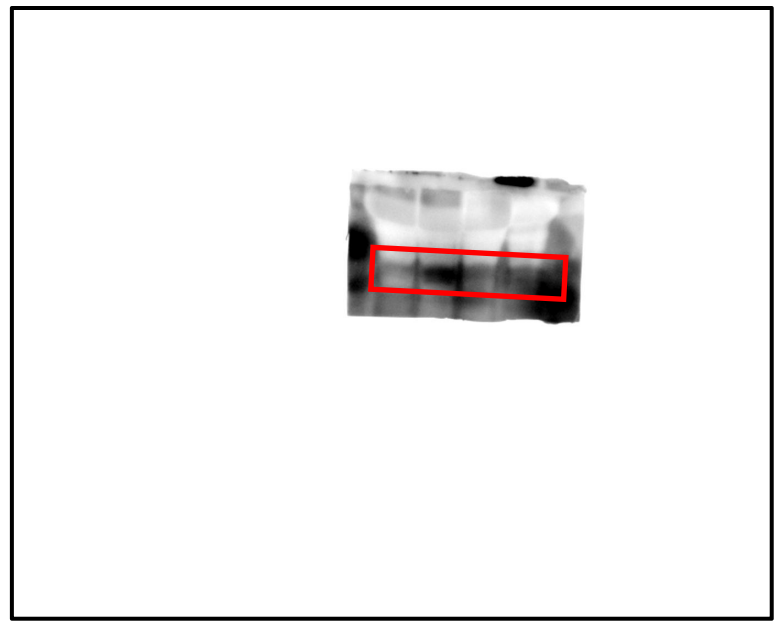

GAPDH

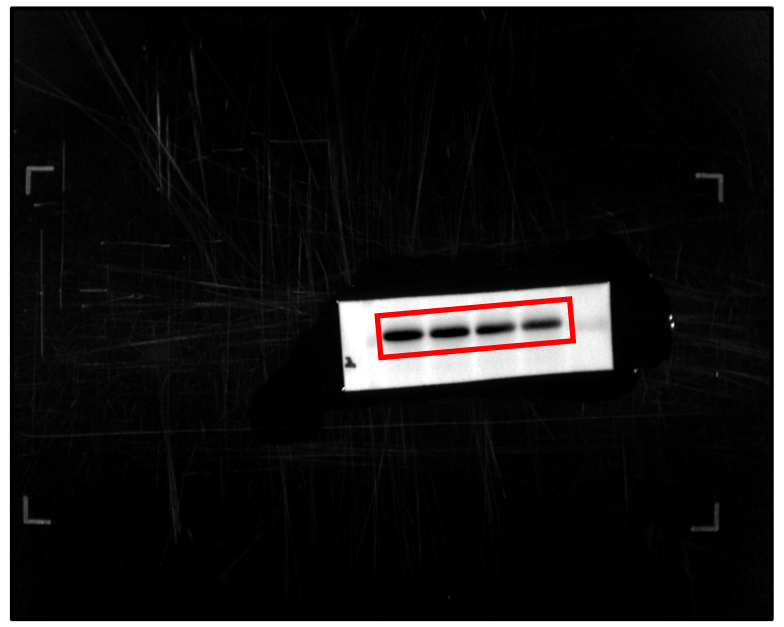

Fig. 8A

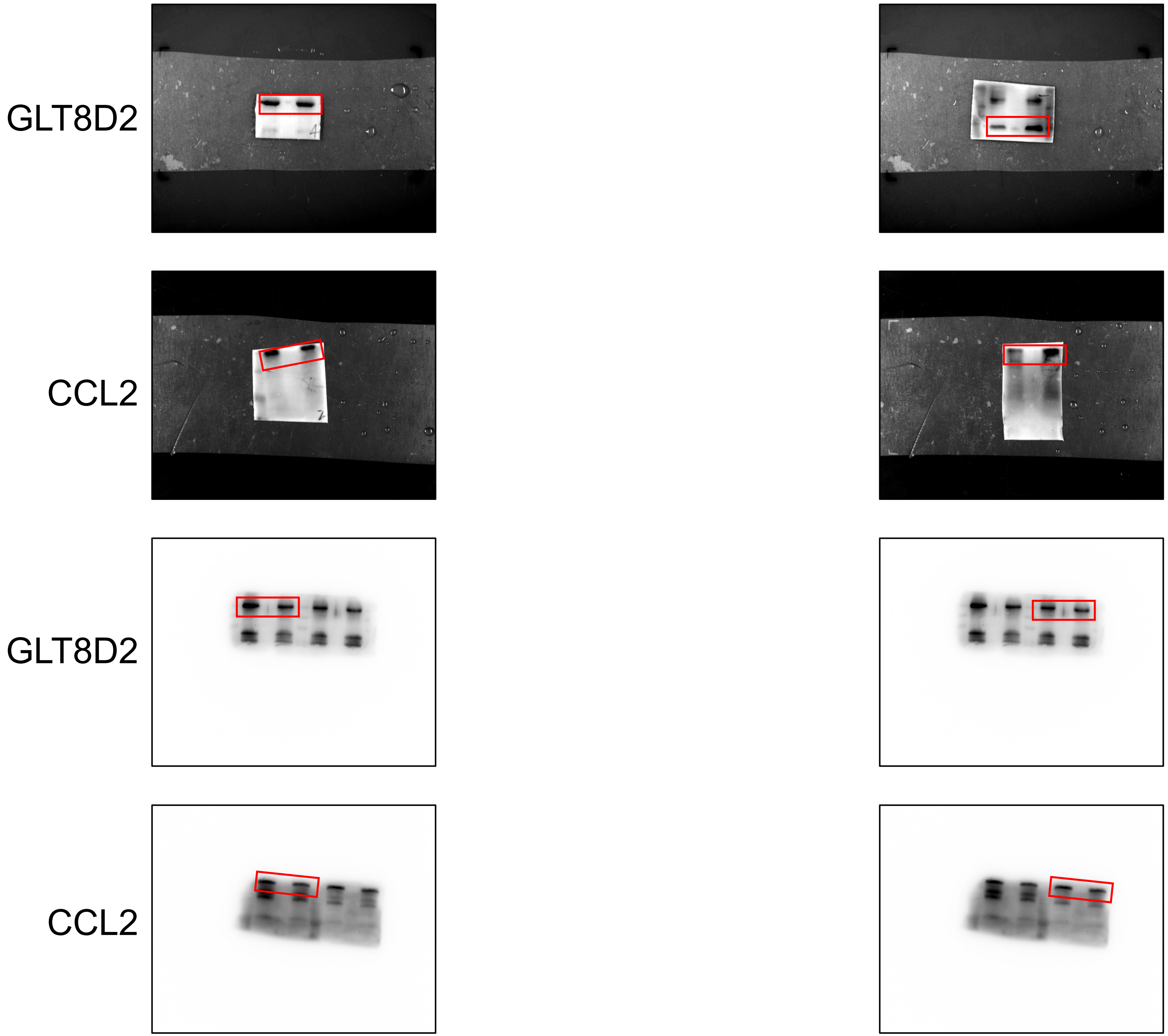

Fig. 8C

CCL2

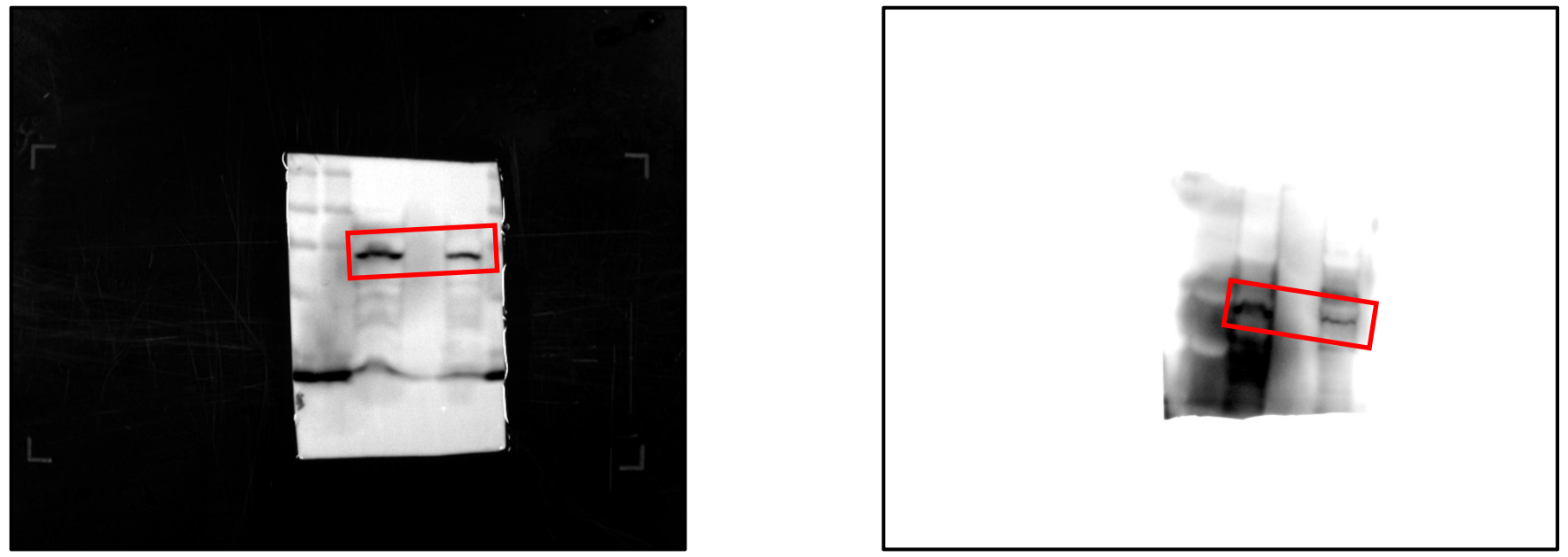

Fig. 8D

CCL2

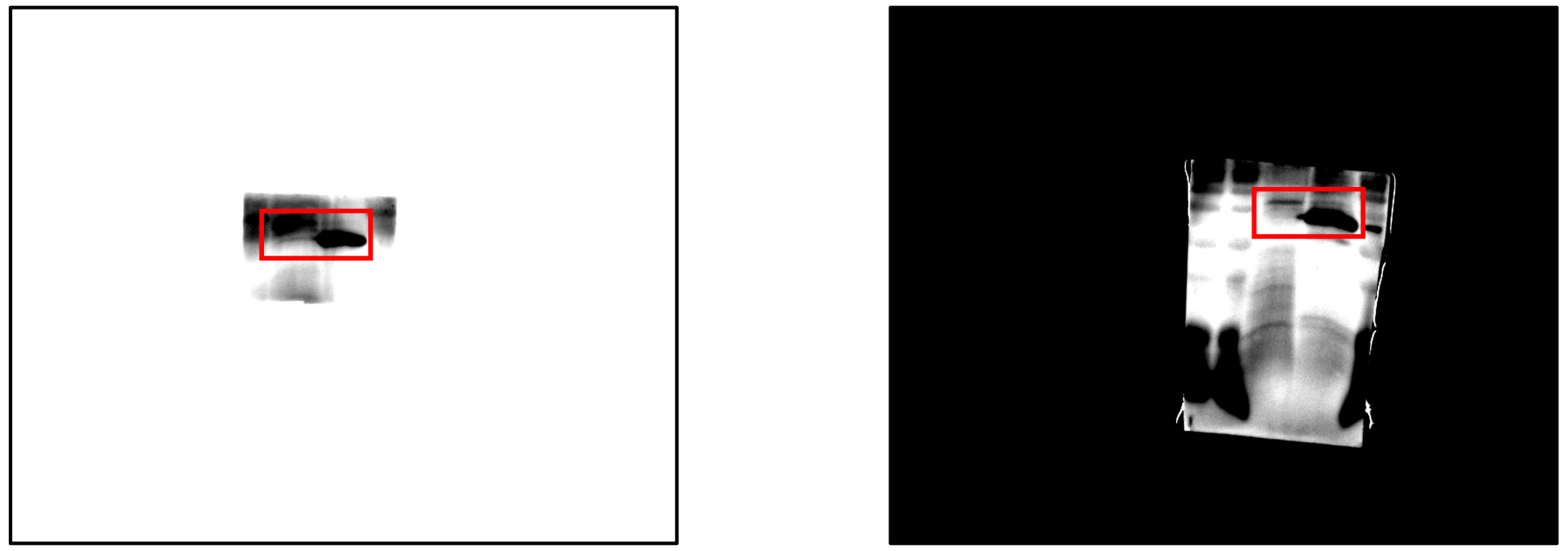

Fig. 8E

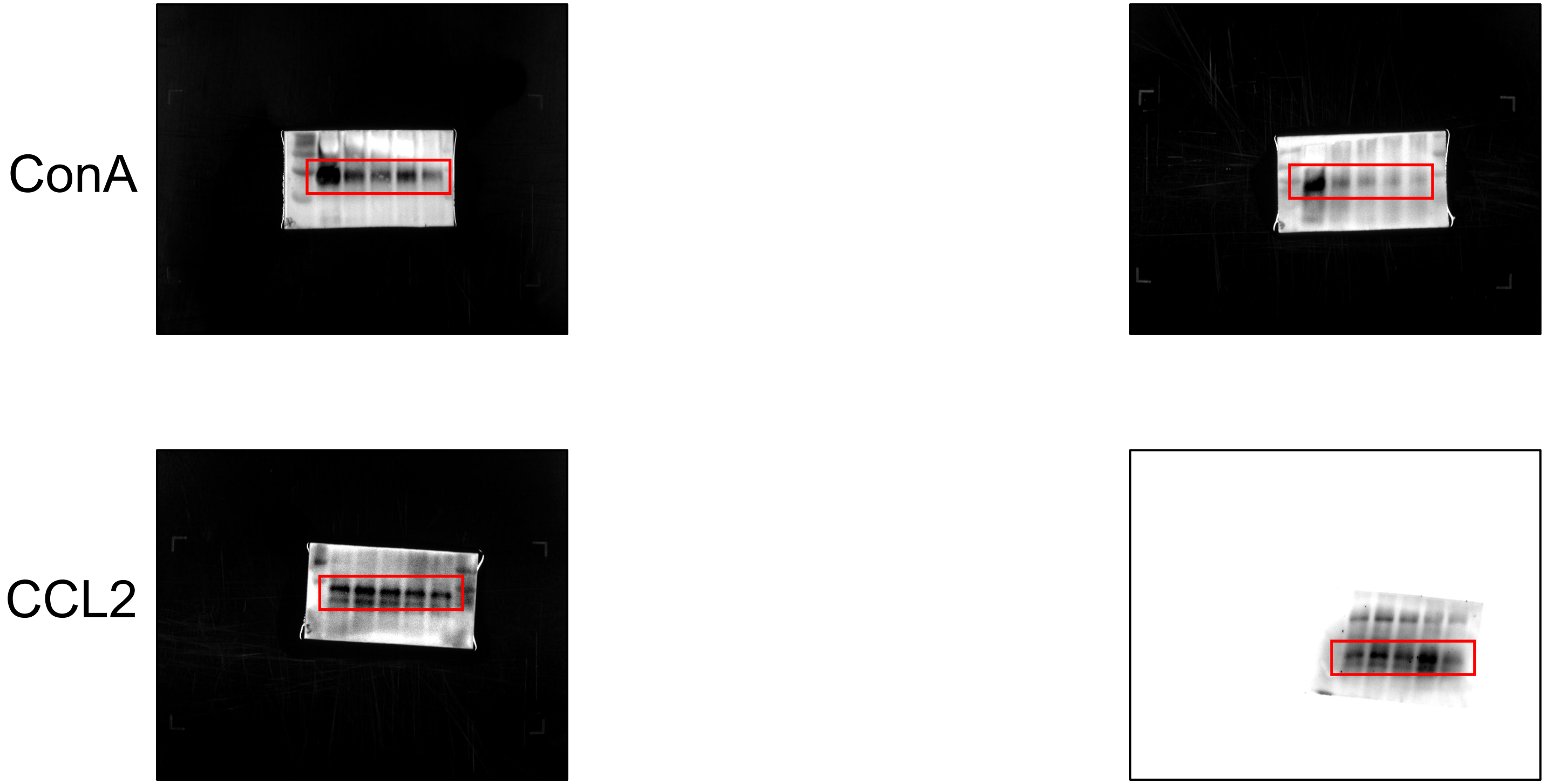

Fig. S1B

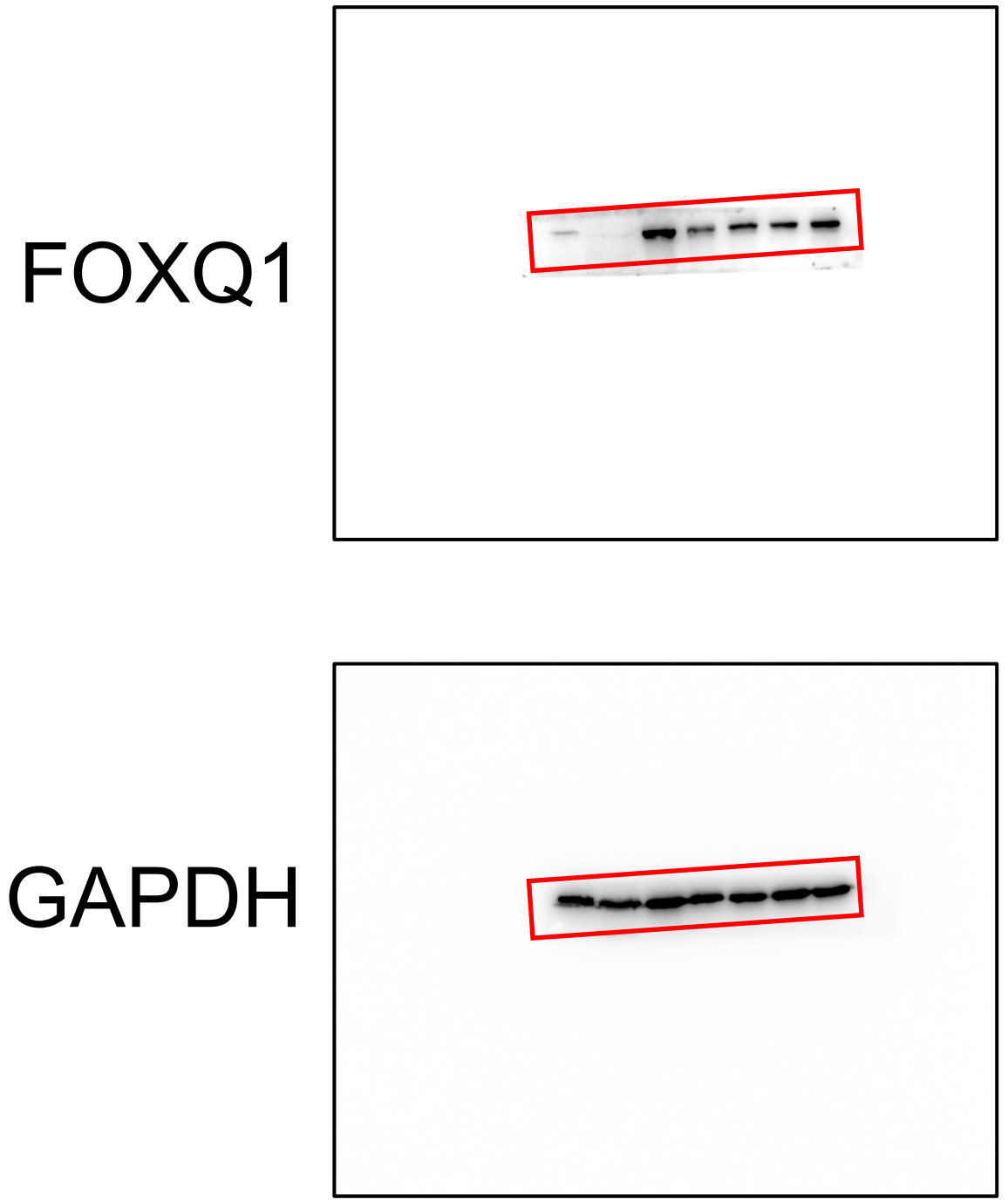

Fig. S1D

FOXQ1

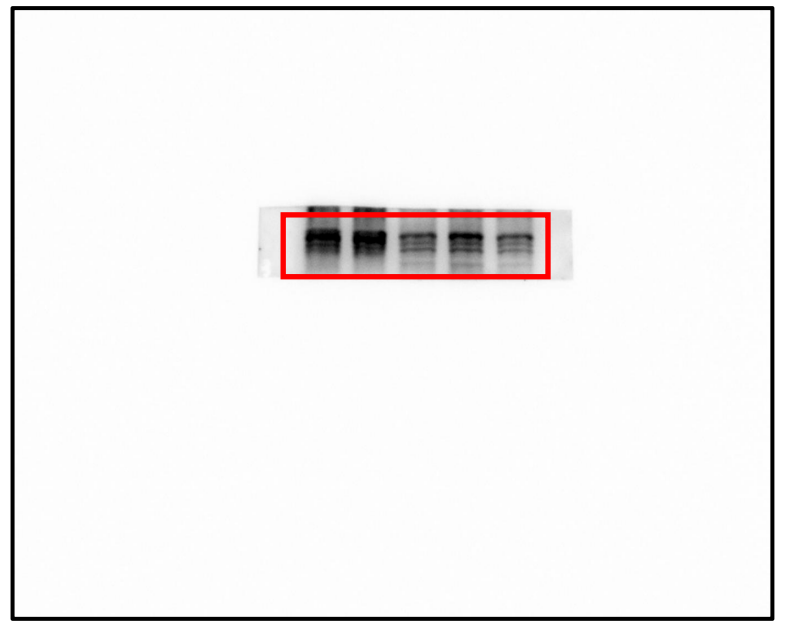

GAPDH

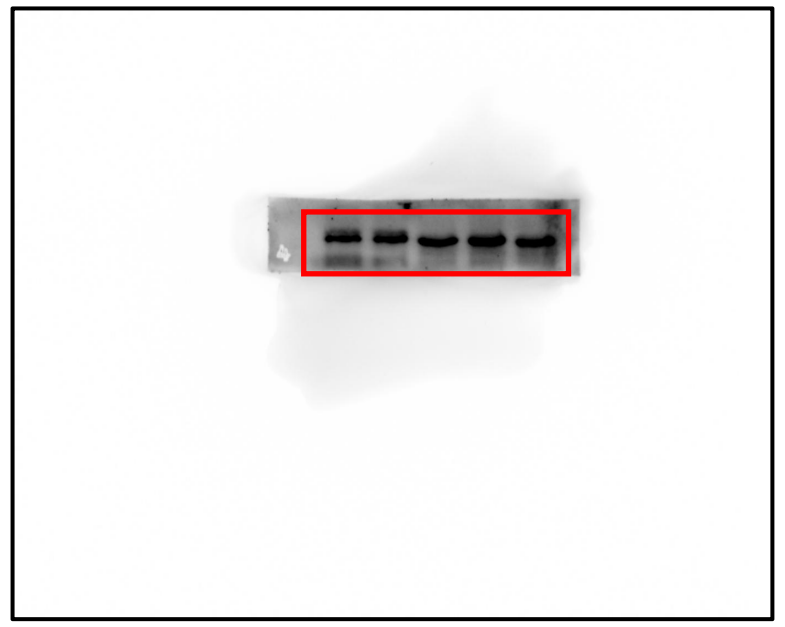

Fig. S1G

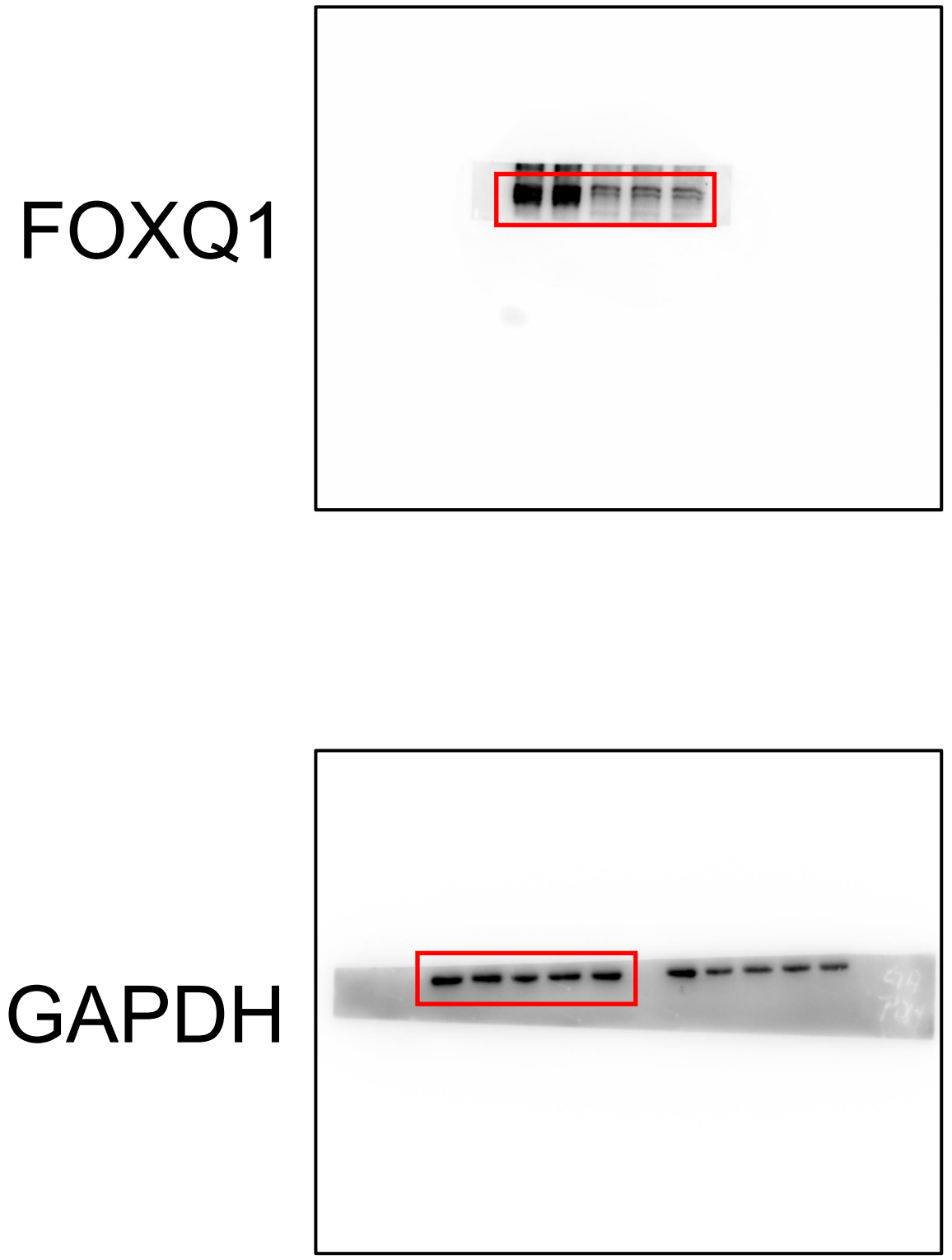

Fig. S1I

FOXQ1

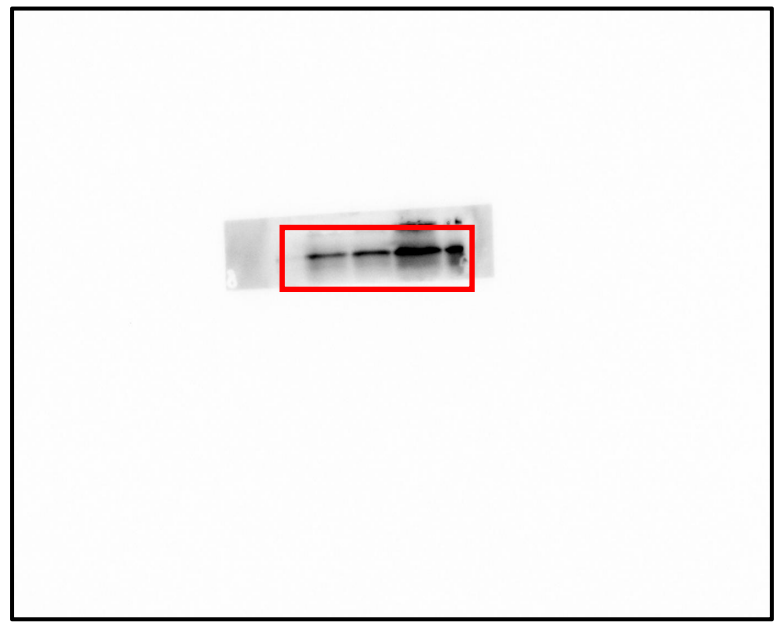

GAPDH

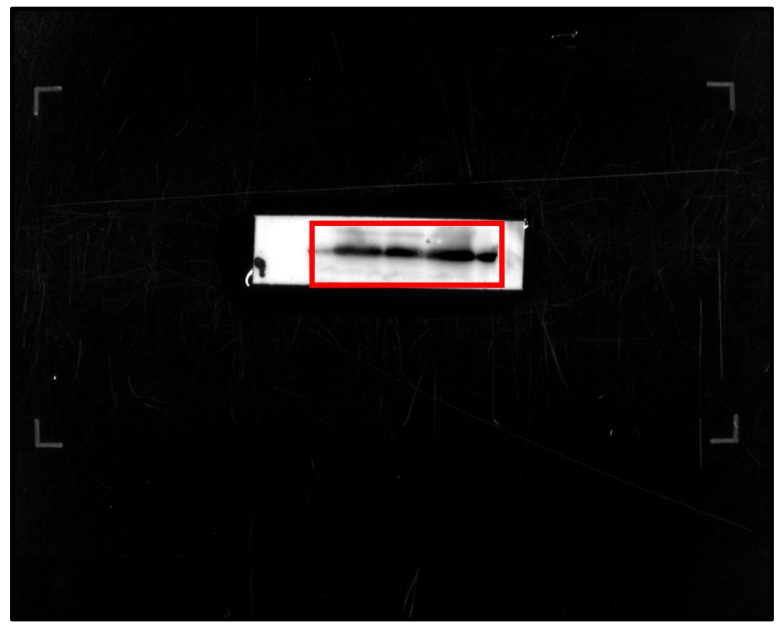

Fig. S5N

GLT8D2

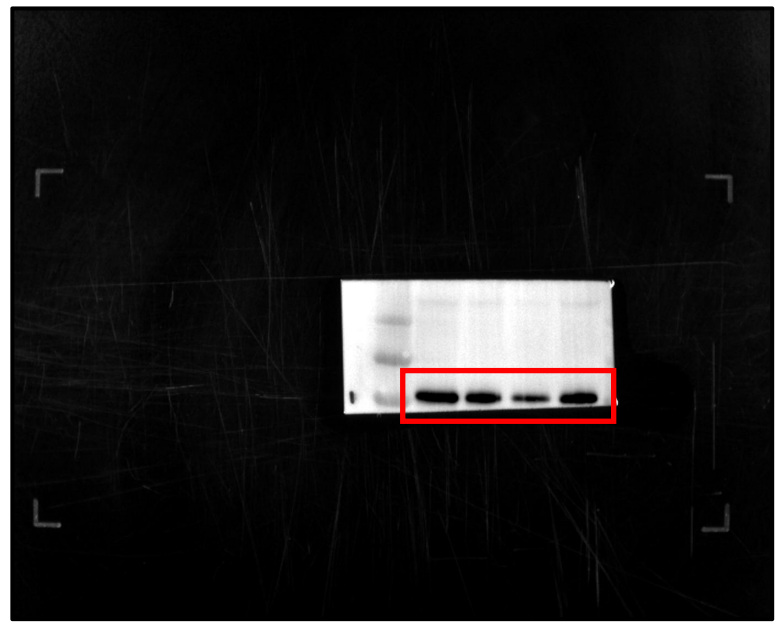

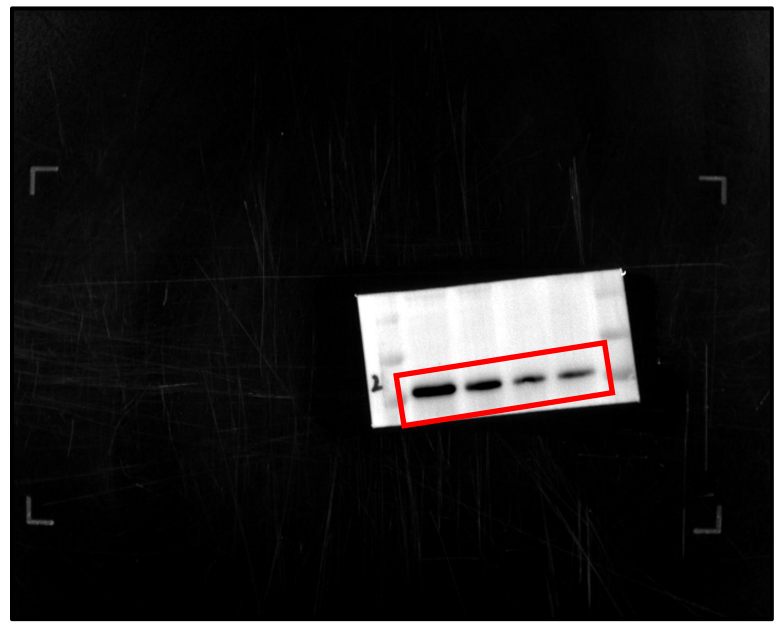

β-Tubulin

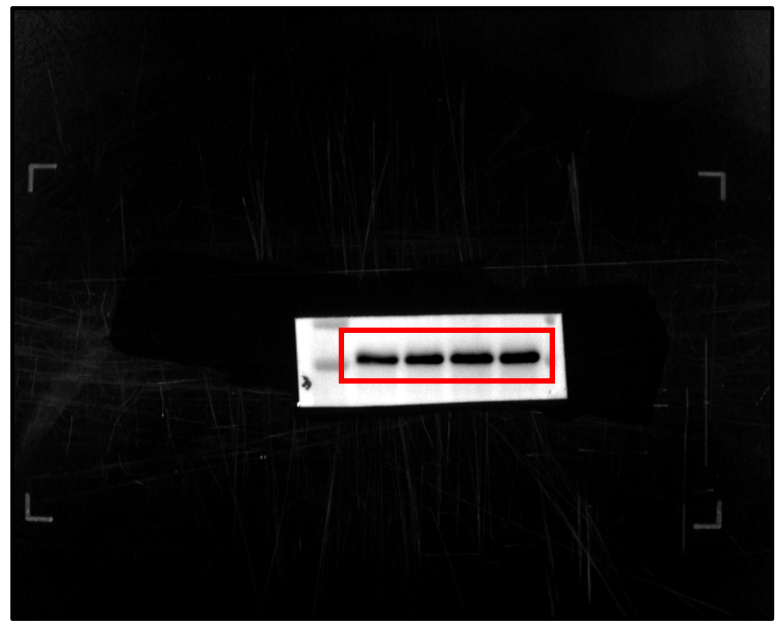

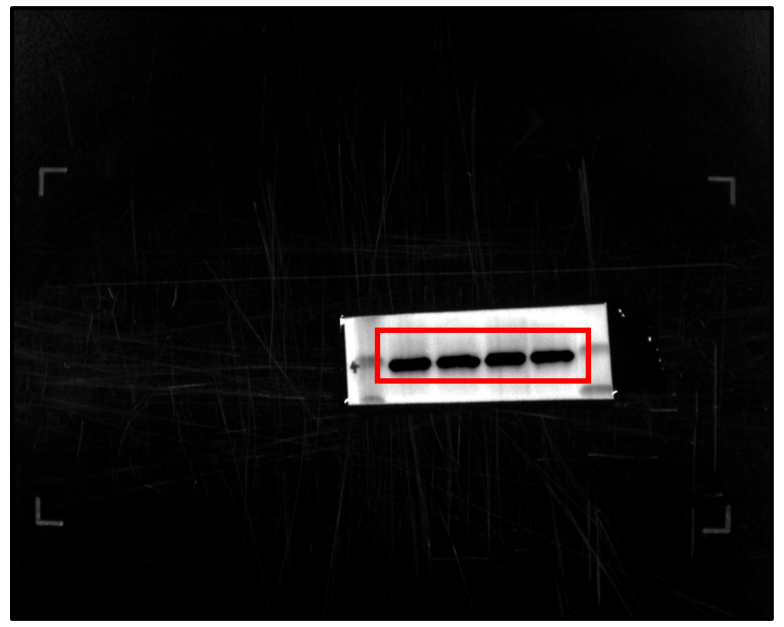

Supplement: Supplementary file 1 [file 41419_2026_9046_MOESM1_ESM.pdf]
