# Supplementary material for: FOXQ1 activates GLT8D2 to enhance CCL2 N-glycosylation and promote prostate cancer bone metastasis
Source: Cell Death Dis. 2026 Jul 1;17(1):817. doi: 10.1038/s41419-026-09046-9 (PMC13590482; doi:10.1038/s41419-026-09046-9)
Supplement: Supplementary file 2 [file 41419_2026_9046_MOESM2_ESM.docx]

**Methods And Materials**

**Immunohistochemistry (IHC)**

IHC was used to examine the expression of FOXQ1 and related proteins in clinical PCa tissues and subcutaneous tumor tissues. FOXQ1 (Proteintech, USA), GLT8D2 (Bioss, China), CCL2 (Proteintech, USA), E-Cadherin (Proteintech, USA), β-catenin (Proteintech, USA), and Vimentin (Proteintech, USA) antibodies were diluted 1:300 with universal antibody diluent (EpiZyme, China) and incubated overnight on tissue sections. Mayer’s hematoxylin was used for nuclear counterstaining. Protein expression was scored using the following criteria: percentage of positively stained cells (0%, 25%, 50%, 75%, 100%) was given scores from 0 to 4, and staining intensity (none, light yellow, dark yellow, dark brown) was scored from 0 to 3. The total staining score was the product of the percentage score and intensity score. For distinguishing high and low expression of FOXQ1 in BPH, primary PCa, and bone metastatic PCa tissues, a FOXQ1 staining score <8 was considered high expression, and ≤8 was low expression. In bone metastatic PCa, FOXQ1 staining scores >10, GLT8D2 and CCL2 staining scores >11 were considered high expression in expression relevance analysis.

**Western Blot/Lectin Blot**

Cell lysates were prepared in RIPA lysis buffer. The following primary antibodies were used: FOXQ1 (BAIJIA, China), GAPDH (Proteintech, USA), E-Cadherin (Abmart, China), β-catenin (Abmart, China), Vimentin (Abmart, China), GLT8D2 (Bioss, China), CCL2 (Proteintech, USA), β-tubulin (Proteintech, USA), all diluted 1:1000 with universal antibody diluent (EpiZyme, China). Lectin blot was performed using ConA (Vector Laboratories, USA) at 1:1000 dilution. ImageJ was used for quantitative analysis of the results.

**Quantitative Reverse Transcription Polymerase Chain Reaction (qRT-PCR)**

Total RNA was extracted using TRIZOL reagent, followed by reverse transcription. qRT-PCR was performed using SYBR Green PCR master mix (Applied Biosystems, USA) on an ABI 7500HT system. Primer sequences are shown in Table S2. GAPDH and *Gapdh* were used as endogenous controls for human and mouse samples, respectively. Relative quantification was calculated using the 2^-ΔΔCT^ method.

**Cell Proliferation Assays**

For clonogenic assays, PCa cells were seeded at 500 cells per well in 6-well plates. After 10 days of culture, cells were fixed with methanol for 15 minutes, stained with 0.1% crystal violet solution for 20 minutes, and colony counts were analyzed using ImageJ.

Cell proliferation was also assessed using the Cell Counting Kit-8 (CCK-8, Dojindo, Japan). PCa cells were seeded at 1000 cells per well in 96-well plates, and cell viability was measured every 24 hours according to the manufacturer’s instructions.

EdU incorporation assays were performed using the EdU proliferation kit (MeilunBio, China). PCa cells were seeded at 1×10^5^ cells per well in 6-well plates and incubated with 100 μM EdU for 2 hours. Cells were visualized under a fluorescence microscope.

**Flow Cytometry Analysis**

Cell cycle and apoptosis assays were performed using kits from KeyGEN (China) and Vazyme (China). Anti-Hu CD68, Anti-Hu CD163, Anti-Mouse CD11b, and Anti-Mouse CD163 antibodies (Invitrogen, USA) were used at a 1:200 dilution and incubated at room temperature for 30 minutes.

**Cell Migration Assays**

For wound healing assays, PCa cells were seeded at 4×10^5^ cells per well in 24-well plates and grown to confluence. A scratch was made using a 200 μL pipette tip, and migration was observed with fresh medium.

In transwell assays, 1×10^5^ cells were resuspended in 200 μL medium and placed in the upper chamber of an 8.0 μm transwell chambers (BD, USA). In the migration assay, the lower chamber contained 600 μL complete medium, while for macrophage migration, the lower chamber contained 600 μL macrophage recruitment co-culture medium.

**Chromatin Immunoprecipitation (ChIP)**

The Sonication ChIP Kit and AFTSpin Multifunction DNA Purification Kit were purchased from Abclonal (China). The FOXQ1 antibody (5 μg, Proteintech, USA) was prepared in the incubation solution. The GLT8D2 promoter was divided into 9 motifs, and qRT-PCR primers for each motif were designed by Sango (China) and synthesized by Tsingke Biotech (China). The primer sequences are displayed in Table S2. The qRT-PCR products were analyzed by nucleic acid gel electrophoresis.

**Co-immunoprecipitation (Co-IP)**

Protein A/G magnetic beads, purchased from Selleck (USA), were used in this study. The primary antibodies for CCL2 (4 μg, Proteintech, USA) and GLT8D2 (4 μg, Bioss) were prepared at the same concentration for the incubation solution. ConA magnetic beads were purchased from Beyotime (China).

**Confocal Immunofluorescence**

PCa cells were seeded into confocal dishes and cultured for 24 hours. ConA (1:400, Vector Laboratories, USA) and CCL2 (1:200, Proteintech, USA) were used as primary antibodies and incubated overnight. Confocal immunofluorescence microscopy was employed to observe the fluorescence signals.

**Dual-Luciferase Reporter Assay**

The dual-luciferase reporter plasmids were designed and constructed by Tsingke Biotech (China). The dual-luciferase reporter gene detection kit was purchased from Promega (USA). Plasmids were transfected into PCa cells using Lipofectamine 3000 (Invitrogen, USA), and luciferase activity was measured according to the manufacturer's instructions.

**Protein Assay using Cell Supernatant**

A total of 5 × 10⁶ PCa cells were cultured in complete medium for 8 hours until they fully adhered, followed by a 24-hour incubation in serum-free medium. The cell supernatant was collected and proteins were extracted using methanol and chloroform for Western blot analysis, or alternatively, Enzyme-Linked Immunosorbent Assay (ELISA) was used for detection. The CCL2 ELISA kit was purchased from FineTest (China).

**Table S1 Target sequences of siRNAs and shRNAs**

| **Name** | **Target sequence (5‘-3’)** |
| --- | --- |
| si1FOXQ1 | GCAACGGGCUACAGCUUUA |
| si2FOXQ1 | GCUAUUGACCGAUGCUUCA |
| si3FOXQ1 | GAACCUUUCCACACUAUAA |
| sh1FOXQ1 | GCTGGCGGAGATCAACGAGTA |
| sh2FOXQ1 | CGAGTACCTCATGGGCAAGTT |
| sh3FOXQ1 | GTGCACGCAGCAAGCCATATA |
| sh1GLT8D2 | GCCCAAGAATGACGCAGATGA |
| sh2GLT8D2 | GCAACACTGACGCCAACATCT |
| sh3GLT8D2 | GCAGGGATATTTAAACTCAAT |

**Table S2 Primers for qRT-PCR**

| **Name** | **Primer（5‘-3’）** |
| --- | --- |
| FOXQ1-F | ATTTCTTGCTATTGACCGATGC |
| FOXQ1-R | CCCAAGGAGACCACAGTTAGAG |
| GAPDH-F | CAGTCAGCCGCATCTTCTT |
| GAPDH-R | GACAAGCTTCCCGTTCTCAG |
| BAX-F | GAGCAGCCCAGAGGCG |
| BAX-R | GGACGCATCCTGAGGCAC |
| BCL2-F | AAAAATACAACATCACAGAGGAAGT |
| BCL2-R | CCTTGGCATGAGATGCAGGA |
| E-Cadherin-F | GCCCTGCCAATCCCGATGAAA |
| E-Cadherin-R | GGGGTCAGTATCAGCCGCT |
| β-catenin-F | GCTTTCAGTTGAGCTGACCA |
| β-catenin-R | CAAGTCCAAGATCAGCAGTCTC |
| Vimentin-F | TGAGTACCGGAGACAGGTGCAG |
| Vimentin-R | GGAGCCACTGCCTTCATAGTCAA |
| Snail-F | CCTGCGTCTGCGGAACCTG |
| Snail-R | GTTGGAGCGGTCAGCGAAGG |
| Slug-F | TCGGACCCACACATTACC |
| Slug-R | CAGATGAGCCCTCAGATTTG |
| *Cstk*-F | AGGGCCAACTCAAGAAGAAAACT |
| *Cstk*-R | TGCCATAGCCCACCACCAACACT |
| *Trap*-F | CGACCATTGTTAGCCACATACG |
| *Trap*-R | TCGTCCTGAAGATACTGCAGGTT |
| *c-Src*-F | TCCTTTGGGATTCTGCTGAC |
| *c-Src*-R | CCAGCACTGGCACATAAGG |
| *Gapdh*-F | CCCCCAATGTATCCGTTGTG |
| *Gapdh*-R | TAGCCCAGGATGCCCTTTAGT |
| GLT8D2-motif1-F | TGACTAGTGCAACTGAGGAACTG |
| GLT8D2-motif1-R | CCAGGTTGGTCCAAATGTACTTAAAAG |
| GLT8D2-motif2-F | GAATTGAGATGTGCTCCAAGTGTAC |
| GLT8D2-motif2-R | CTCCCTTCCTCCTGCCCTTC |
| GLT8D2-motif3-F | GAGGAATAGGTTCTAGTGTAGTATGGC |
| GLT8D2-motif3-R | GGAAACATTCAATATCCTCCTTCTAGC |
| GLT8D2-motif4-F | TAAGCATCAATATTTGAGATTATAGATATG |
| GLT8D2-motif4-R | TAATTTAAAATTTTCTAGTAGCCACATTAA |
| GLT8D2-motif5-F | AGCCTGAGTAACAGAGCAAGACC |
| GLT8D2-motif5-R | CCGCCTCAGCCTCCCAAAG |
| GLT8D2-motif6-F | AGATCTCATCACTGCACTCTAGCC |
| GLT8D2-motif6-R | TTTCTAAAGTGGTGCTCTCCAACAG |
| GLT8D2-motif7-F | GGAAATAGGAAGAGGAGCCATTGC |
| GLT8D2-motif7-R | CTGTTCTGTATCTCTGCGGTGTG |
| GLT8D2-motif8-F | TGGTGTTCTTTCAAAGTTTGCCTTC |
| GLT8D2-motif8-R | ACAACGCTGTTCTGGAGGAAAAG |
| GLT8D2-motif9-F | CAACGCAAGCAAAGGGGAGTG |
| GLT8D2-motif9-R | CTGCTTCGGCTGCTTCTAAGATC |

**Fig. S1 FOXQ1 promotes PCa proliferation and inhibits apoptosis. (A)** The expression of FOXQ1 in normal prostate cell lines and PCa cell lines assessed by qRT-PCR. **(B)** The expression of FOXQ1 in normal prostate cell lines and PCa cell lines evaluated by Western blot. **(C)** The efficiency of siRNA-mediated transient knockdown of FOXQ1 in PC3 cells was assessed by qRT-PCR. **(D)** The efficiency of stable FOXQ1 knockdown in PC3 cells via lentivirus was determined by Western blot. **(E)** The efficiency of stable FOXQ1 knockdown in PC3 cells via lentivirus was assessed by qRT-PCR. **(F)** The efficiency of siRNA-mediated transient knockdown of FOXQ1 in DU145 cells was assessed by qRT-PCR. **(G)** The efficiency of stable FOXQ1 knockdown in DU145 cells via lentivirus was determined by Western blot. **(H)** The efficiency of stable FOXQ1 knockdown in DU145 cells via lentivirus was assessed by qRT-PCR. **(I)** The efficiency of stable FOXQ1 overexpression in C4-2 cells via lentivirus was determined by Western blot. **(J)** The efficiency of stable FOXQ1 overexpression in C4-2 cells via lentivirus was assessed by qRT-PCR. **(K)** Based on the TCGA database, the correlation between FOXQ1 expression and cell cycle proteins in PCa tissues was analyzed using GEPIA. **(L)** After transient FOXQ1 knockdown, the expression levels of BAX and BCL2 in PC3 and DU145 cells were measured by qRT-PCR. ***P < 0.001, **P < 0.01, *P < 0.05, ns indicates P≥0.05.

**Fig. S2 FOXQ1 promotes PCa migration. (A)** Based on the TCGA database, the correlation between FOXQ1 expression and the expression of EMT-related proteins in PCa tissues was analyzed using GEPIA. **(B)** The expression levels of E-Cadherin, β-catenin, Vimentin, Snail, and Slug in PC3 and DU145 cells with transient FOXQ1 knockdown, and in C4-2 cells with stable FOXQ1 overexpression, were assessed by qRT-PCR. ***P < 0.001, **P < 0.01, *P < 0.05, ns indicates P≥0.05.

**Fig. S3 FOXQ1 regulates the PCa tumor microenvironment. (A)** The correlation between FOXQ1 expression and macrophage infiltration in PCa tissues was analyzed using TIMER. **(B)** PCA dimensionality reduction was performed on GSE193337 to identify marker genes. **(C)** Marker genes were identified using CellMarker 2.0. **(D)** Samples were grouped based on high and low FOXQ1 expression in the tumor. **(E, F)** FOXQ1 expression levels in tumor cells from FOXQ1-high and FOXQ1-low groups. **(G)** Cell subgroup analysis was performed for FOXQ1-high and FOXQ1-low groups. **(H, I)** The differences in cell communication between FOXQ1-high and FOXQ1-low groups were studied. **(J)** Based on the TCGA database, the correlation between FOXQ1 expression and M2 macrophage-related proteins in PCa tissues was analyzed using GEPIA. **(K)** The effect of FOXQ1 knockdown in PC3 cells on the recruitment of U937-M0 (U937 cells induced with 100nM PMA for 48 hours) and mouse BMM by cell culture supernatant was investigated. **(L)** The impact of FOXQ1 knockdown in PC3 cells on the polarization of BMM induced by cell culture supernatant was studied. **(M)** Based on the TCGA database, the correlation between FOXQ1 expression and osteoclast-related proteins in PCa tissues was analyzed using GEPIA. **(N, O)** The correlation between FOXQ1 and CCL2 expression in PCa tissues was analyzed using TIMER and GEPIA. The scale bar represents 50 μm. ***P < 0.001, **P < 0.01, *P < 0.05, ns indicates P≥0.05.

**Fig. S4 GLT8D2 is involved in regulating PCa progression. (A)** The correlation between the expression of GLT8D2 and CCL2 in PCa was analyzed using TIMER. Based on the TCGA database, the correlation between the expression of FOXQ1 and GLT8D2 **(A)**, and between GLT8D2 and CCL2 **(C)** in PCa tissues was analyzed using GEPIA. **(D)** The correlation between GLT8D2 expression and macrophage infiltration in PCa tissues was analyzed using TIMER. Based on the TCGA database, the correlation between GLT8D2 expression and EMT-related proteins **(E)**, M2 macrophage-related proteins **(F)**, and osteoclast-related proteins **(G)** in PCa tissues was analyzed using GEPIA.

**Fig. S5 GLT8D2 is involved in regulation of PCa bone metastasis. (A)** PCA dimensionality reduction was performed on the single-cell sequencing data from GSE143791. **(B)** Cell clusters were labeled using CellMarker 2.0. **(C)** Samples were grouped based on high and low GLT8D2 expression in the tumor. (D) Monocytes, macrophages, osteoclasts, and tumor cells were selected and subjected to PCA dimensionality reduction analysis. **(E)** Cell subgroup analysis was performed for GLT8D2-high and GLT8D2-low groups. **(F)** Pathway enrichment heatmap of pseudotime analysis. **(G)** Pseudotime analysis branch plot. **(H)** Pseudotime branch plot showing different cell populations. **(I)** Pseudotime branch plot based on cell state classification. **(J)** Continuous cell pseudotime branch plot. **(K, L)** The differences in cell communication between GLT8D2-high and GLT8D2-low groups were studied. **(M)** GO_CC analysis of the enrichment of upregulated genes in tumor cells from the GLT8D2-high group relative to the GLT8D2-low group, in terms of cellular components. **(N)** The efficiency of GLT8D2 knockdown in PC3 and DU145 cells via lentivirus was determined by Western blot. **(O)** The efficiency of siRNA-mediated transient knockdown of *Ccr2* in RAW264.7 cells was assessed by qRT-PCR. ***P < 0.001, **P < 0.01, *P < 0.05, ns indicates P≥0.05.
